# Syringeal vocal folds do not have a voice in zebra finch vocal development

**DOI:** 10.1038/s41598-021-85929-5

**Published:** 2021-03-19

**Authors:** Alyssa Maxwell, Iris Adam, Pernille S. Larsen, Peter G. Sørensen, Coen P. H. Elemans

**Affiliations:** Department of Biology, University of Southern Denmark, 5230 Odense, Denmark

**Keywords:** Neuroscience, Physiology, Zoology

## Abstract

Vocal behavior can be dramatically changed by both neural circuit development and postnatal maturation of the body. During song learning in songbirds, both the song system and syringeal muscles are functionally changing, but it is unknown if maturation of sound generators within the syrinx contributes to vocal development. Here we densely sample the respiratory pressure control space of the zebra finch syrinx in vitro. We show that the syrinx produces sound very efficiently and that key acoustic parameters, minimal fundamental frequency, entropy and source level, do not change over development in both sexes. Thus, our data suggest that the observed acoustic changes in vocal development must be attributed to changes in the motor control pathway, from song system circuitry to muscle force, and not by material property changes in the avian analog of the vocal folds. We propose that in songbirds, muscle use and training driven by the sexually dimorphic song system are the crucial drivers that lead to sexual dimorphism of the syringeal skeleton and musculature. The size and properties of the instrument are thus not changing, while its player is.

## Introduction

Many vertebrates change their vocal output over postnatal development^1–4^. Especially in species capable of vocal imitation learning, progressive changes in vocal output are attributed to changes in the underlying brain circuitry. However, the maturation of the vocal organ and tract have profound effect on voice during puberty in humans^5^, and can explain transitions from juvenile to adult calls in marmosets^6^. Thus, the postnatal development of the body can drive changes in vocal behavior, and therefore needs to be included when studying vocal development.

The zebra finch is a widespread animal model to study the mechanisms underlying vocal imitation learning and production^7,8^. In male zebra finches, vocalizations change dramatically in structure and variability from the onset of song learning at 25 DPH to adulthood^2,3,9^. Song develops from variable subsong (~ 25 DPH) to plastic song (50 DPH) to highly stereotyped crystallized song (100 DPH)^9^. These changes, including the ones in acoustic features within syllables, such as fundamental frequency (*f*_o_), amplitude and entropy, are typically attributed to changes in neural circuitry.

However, the vocal organ, the syrinx, is also undergoing significant anatomical and functional changes during song development^10–13^. Coinciding with the transition from the sensory to the sensorimotor phase of learning around 20 DPH, syringeal muscles in male zebra finches increase in mass and cross-sectional area^10^. Additionally, the contractile properties of male syringeal muscles increase in speed^11^, which affects the transformation from neural commands to muscle force^13^. Thus, over development functional changes occur in the syrinx that change the transformation from neural signals into force which thereby change motor control of the vocal organ.

**Figure 1:**
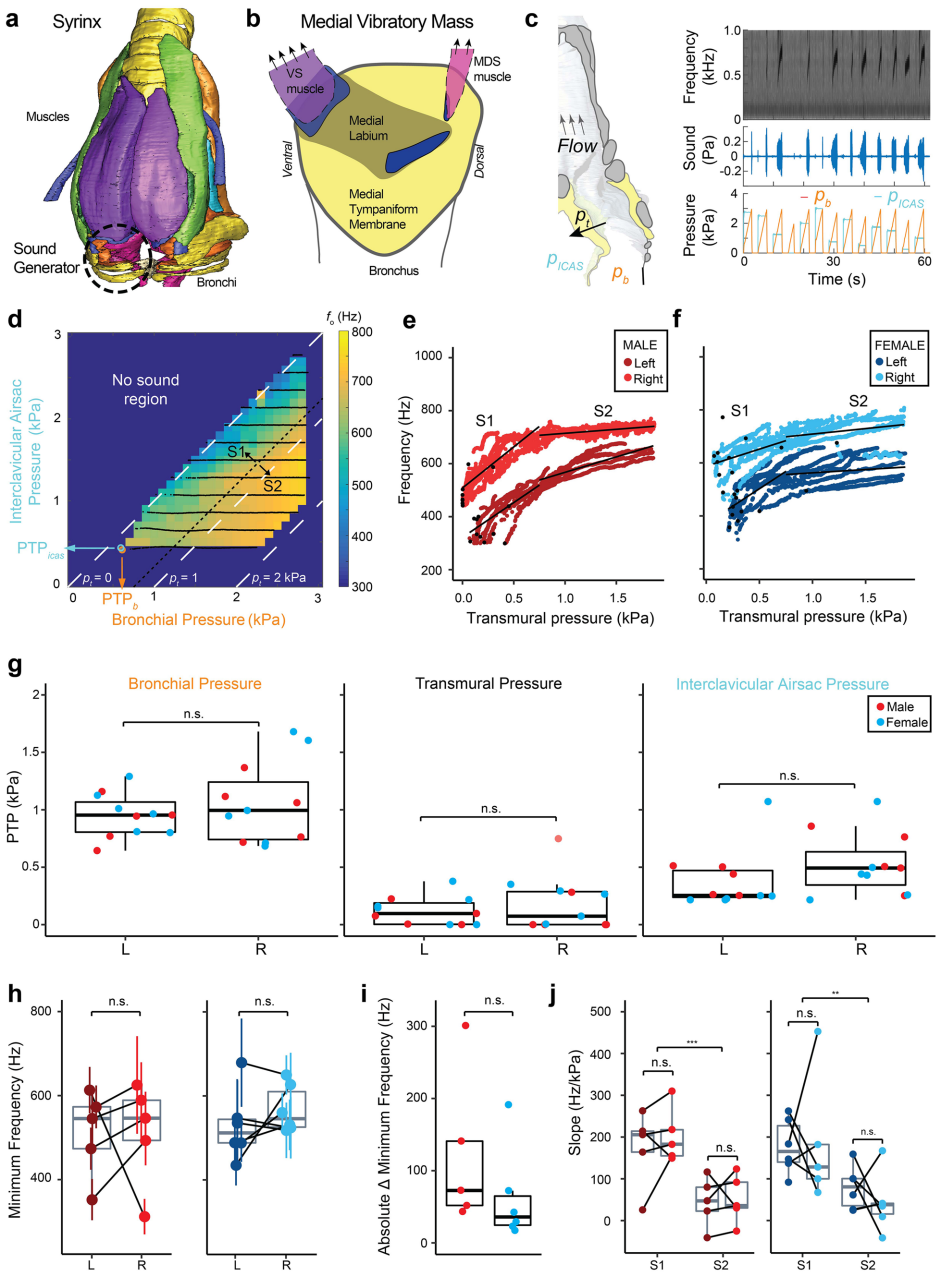
The adult zebra finch syrinx produces sound in a well-defined pressure control space with higher frequencies on the right. (**a**) Ventral view of the male syrinx. One of the two sound generators are indicated by a dashed circle. Image based on ref.^20^. (**b**) Schematic view on the sound generator’s medial vibratory mass (MVM). (**c**) Schematic sagittal cross-section through the syrinx of the left bronchus, indicating the relevant pressures. Example raw data of sound production induced by bronchial (orange) and interclavicular air sac (light blue) pressures (**d**) Example of fundamental frequencies produced in the pressure control space for the right hemi-syrinx of an adult male zebra finch (100 DPH). Phonation threshold pressures (PTP) are indicated for: PTP_icas_ (light blue arrow), and PTP_b_ (orange arrow). Transmural pressure (*p*_t_) is indicated by white dashed lines for *p*_t_ = 0, 1 and 2 kPa. (**e**,**f**) Frequency increases with *p*_t_ for both the left (dark red, dark blue) and right hemi-syrinx (light red, light blue) in (**e**) males and (**f**) females, respectively. Black lines indicate linear regressions for region S1 (0 < *p*_t_ < 0.75) and S2 (*p*_t_ > 0.75). The phonation threshold pressure (PTP) values for individual *p*_icas_ ramps are indicated by black circles. (**g**) PTP for *p*_b_, *p*_t_, and *p*_icas_ for the left and right hemi-syrinx. (**h**) Minimum frequency produced by the right hemi-syrinx was higher than the left hemi-syrinx (p = 0.09, see Table S1) for most individuals. (**i**) The absolute difference of minimal *f*_o_ was not significantly different between males and females. (**j**) Slopes for the left and right hemi-syrinx did not differ significantly between sides but were significantly steeper in S1 than in S2. For statistics see Supplementary Table S1. *p < 0.05; **p < 0.01; ***p < 0.001. Images created with Adobe Illustrator CS6.

It is currently unknown if the syrinx exhibits changes over song development that can affect the complex transformation from muscle forces to sound. Analogous to mammals, birds produce sound when expiratory airflow from the bronchi induces self-sustained vibrations of vocal fold-like structures, the labia, within the syrinx^14–17^ (Fig. 1a) following the myoelastic-aerodynamic (MEAD) theory for voice production^18^. Songbirds have two bilateral sets of independently controlled paired folds or labia, one in each bronchus or hemi-syrinx, with the lateral labium on the lateral side, and the medial vibratory mass (MVM) on the medial side. The MVM is a tissue continuum in which the thicker part is called the medial labium (ML)^19–21^ (Fig. 1b). While in most songbird species investigated these labia consist of multiple tissue layers, in zebra finches they seem rather homogeneous in the distribution and orientation of collagen and elastin fibers^21,22^. According to MEAD, acoustic features, such as source level (SL) and *f*_o_, or boundary conditions for vibration, such as the phonation threshold pressure, are largely set by the positioning and tension of, and driving pressures^18^ on, the vocal folds, as well as their resonance properties and shape. Vocal fold resonance properties in turn are determined by vocal fold length and structure, such as collagen and elastin fiber composition, the occurrence of tissue layering and layer orientation^23^. Postnatal changes in size, structure and mechanical properties of the vocal folds in humans^24–26^, and marmosets^6^ can cause dramatic changes in vocal output over vocal development. Thus, material property changes of syringeal MVM could also contribute to changes in vocal output in songbirds.

In zebra finches, surprisingly we have no information on either basic morphology, material properties or acoustic output of the MVM as a function of postnatal development. To our best knowledge syrinx developmental information seems missing for any songbird species. To isolate the contribution of the MVM to the transformation from neural commands to sound, MVM histology^21,22^ or mechanical properties, such as tensile tests^27^, would provide important information, but do not provide the sound ultimately produced. Sound pressure waveforms or features can be predicted using computational models that require tissue property information. For example, one-dimensional string models can coarsely predict *f*_o_ or *f*_o_-ranges from tissue properties^27,28^, but not an exact *f*_o_ resulting from a specific muscle length or respiratory pressure amplitude, nor other acoustic parameters. Full complexity 3D fluid–structure-acoustic-interaction models can predict time-resolved pressure waveforms^29^. A recent model of the (much less complex) pigeon syrinx predicted several acoustic parameters rather accurately based on geometry and material properties^15^, but we do not have such a model for the songbird syrinx. However, in either case, models produce mere predictions that need to be tested against experimental data to validate their accuracy. Thus, we aim at measuring sound output of the vocal organ, and not proxies thereof, as a function of driving parameters.

Furthermore, measuring acoustic features, such as *f*_o_, SL, harmonic content, or entropy during song over development cannot provide conclusive data on the contribution of a changing transformation from muscle to sound. First, during trial-and-error learning, individuals are correcting their song for deviations from the targets^30,31^. Thus, when the acoustic output of the vocal organ or its control space would be changing, the individual would correct for the resulting error. By looking at in vivo singing behavior we cannot exclude compensatory correction for changes driven by tissue maturation. Therefore, we need precise experimental control over driving parameters.

Second, in many songbird species the left and right hemi-syrinx contribute differently to the total frequency range^32–34^. Simultaneous air flow through the left and right hemi-syrinx indicates that some syllables are produced by bilateral oscillations in several songbird species^35,36^, including zebra finches^37,38^. When both sides oscillate independently this would give rise to two independent frequencies^32–34^. This phenomenon is one of the salient features of birdsong. However, other examples show that only one fundamental frequency is produced during simultaneous air flow through the left and right hemi-syrinx, where flow oscillations can be in or out of phase between sides^38^. When two mechanical oscillators have similar resonance frequencies and are physically close they can become mechanically coupled by energy injections^39,40^. Such injection locking, or injection pulling, can force one oscillator to oscillate at the resonance frequency of the other depending on the coupling strength. Because the two syrinx sides are physically close, we cannot exclude that the two sides become mechanically coupled by injection locking if the two sides have tissue resonance frequencies that are sufficiently similar. Thus, despite different resonance frequencies of left and right MVMs^19^, the two sides can become coupled and oscillate at the same frequency. After bilateral nerve cuts single *f*_o_ harmonic stacks are produced^41–43^, which supports the idea that the two sides are coupled by injection locking. In summary, we cannot exclude that injection locking plays a role in zebra finch song in vivo and thereby cannot conclude from in vivo song if the behavior of one side changes over development. Consequently, to isolate the contribution of the MVM to the transformation from neural commands to sound, we need to start by looking at the contributions of each side individually.

Taken together, to measure the complex transformation from muscle forces to sound, we need to measure the sound produced by each hemi-syrinx, driven by physiologically realistic driving parameters under controlled experimental conditions without compensatory neural control.

Two independent motor systems control the tension and positioning of MVMs in songbirds: the respiratory and the syringeal motor system. The respiratory system generates the bronchial pressure and flow driving MVM oscillations. The syrinx is suspended in a pressurized air sac, the interclavicular air sac (ICAS), that is essential to achieve oscillation and sound production^14,44,45^. Both bronchial and ICAS pressure have been shown to correlate to changes in *f*_o_ during manipulation of *p*_icas_ in vivo^45,46^ and ex vivo^14^. However, we currently lack densely sampled characterization of the pressure control space in the zebra finch syrinx and do not know how both pressures interact to drive acoustic parameters.

The syringeal motor system of songbirds consists of up to 8 pairs of intrinsic and extrinsic syringeal muscles^20,47,48^ (Fig. 1a), which control the positioning of individual cartilages and bones within the syringeal skeleton as well as the torque on them. Hereby syringeal muscles control—either directly or indirectly—the adduction level and tension of the labia, which are suspended within the syringeal skeleton^27^. Labial adduction level and tension provide fine-control of air flow and *f*_o_^49^. The control space of syringeal muscles is vast due to its multidimensionality and our understanding limited^14,48,50^. Both changes in the shape and acoustic features of the motor control space could contribute to vocal development, but this is currently unknown.

Here we test if the complex transformation from driving forces to acoustic output of the zebra finch syrinx changes over vocal development. We designed an experimental paradigm to quantify sound production of the syrinx as a function of physiological driving parameters. In a first step, we did not include the biomechanical effect of syringeal muscles, but focused on syringeal dynamics controlled by respiratory pressures and densely sampled the control space of bronchial versus air sac pressure.

## Materials and methods

### In vitro sound production

#### Animals use and care

Zebra finches (*Taeniopygia guttata,* order Passeriformes) were kept and bred in group aviaries at the University of Southern Denmark, Odense, Denmark on a 12:12 h light:dark photoperiod and given water and food ad libitum. Adult birds were provided with nesting material, ad libitum*.* Nest boxes were monitored daily, so that birds could be accurately aged. Breeding began in December 2017 and ended November 2018.

We studied vocal output of the freshly dissected syrinx in vitro in four age groups: 25 DPH (N = 5 males, N = 5 females), 50 DPH (N = 4 males, N = 4 females), 75 DPH (N = 6 males, N = 4 females), and 100 DPH (N = 5 males, N = 6 females). Sex was determined by dissection postmortem (25 DPH) or plumage (all other ages).

#### Approval for animal experiments

All experiments were conducted in accordance with the Danish law concerning animal experiments. The protocol was approved by the Danish Animal Experiments Inspectorate (Ministry of Environment and Food, Glostrup, Denmark, Journal number: 2019-15-0201-00308). The study was carried out in compliance with the ARRIVE guidelines.

#### Syrinx mounting procedure

Animals were euthanized by Isoflurane (Baxter, Lillerød, Denmark) overdose. The syrinxes were dissected out while being regularly flushed and then submerged in a bath of oxygenated Ringer’s solution (recipe see^51^) on ice. Each syrinx was photographed with a Leica DC425 camera mounted on a stereomicroscope (M165-FC, Leica Microsystems, Switzerland) prior to being placed inside the experimental chamber. Each bronchus was placed over polyethylene tubing (outer diameter 0.97 mm × inner diameter 0.58 mm, Instech Salomon, PA, USA) and secured with 10-0 nylon suture (AROSurgical, Newport Beach, CA, USA). The trachea was placed into a 1 ml pipette which was cut to the length of 15 mm (outer diameter 2.4 mm, inner diameter 1.4 mm). Some adipose tissue was left on the trachea to ensure an airtight connection. The total length from syrinx to tracheal outlet was 35 mm, similar to the upper vocal tract length of 28–33 mm^52^. To control the pressure inside the chamber, it was made airtight with a glass lid, which also allowed visualization of the syrinx through the stereomicroscope.

#### Experimental setup

We designed an experimental setup that allows studying the mechanical behavior of the syrinx in vitro or ex vivo (described in detail in^14^). Both in vitro and ex vivo preparations use fresh, alive tissues. The ex vivo preparation is also perfused through the original vasculature^14^. The bronchial pressure (*p*_b_) and pressure in the experimental chamber (i.e. interclavicular air sac pressure, *p*_icas_) can be controlled independently by two dual-valve differential pressure controllers (model PCD, 0–10 kPa, Alicat Scientific, AZ, USA). Bronchial flow through the syrinx was measured with a MEMS flow sensor (PMF2102V, Posifa Microsystems, San Jose, USA) about 7 cm upstream from the bronchial connection. Sound was recorded with a ½ in. pressure microphone-pre-amplifier assembly (model 46AD, G.R.A.S, Denmark), amplified and high-pass filtered (0.2 Hz, 3-pole Butterworth filter, model 12AQ, G.R.A.S., Denmark). The sound recording sensitivity was tested before each experiment using a 1 kHz tone (sound calibrator model 42AB, G.R.A.S., Denmark). The microphone was placed 15 cm from the tracheal connector outlet and positioned at a 45° angle to avoid the air jet from the tracheal outlet. The sound, pressure and flow signals were low-pass filtered at 10 kHz (model EF120, Thor Labs) and digitized at 50 kHz (USB 6259, 16 bit, National Instruments, Austin, Texas).

#### Experimental protocol

To explore the acoustic output of the avian syrinx (Fig. 1a), we subjected the syrinxes to *p*_b_ ramps from 0 to 3 kPa (over atmospheric pressure) at 1 kPa/s with *p*_icas_ at a constant pressure. In one run, we applied 13 *p*_icas_ settings at equidistant 0.23 kPa intervals between 0 and 3 kPa in a randomized order with 2 s pause (*p*_b_ and *p*_icas_ = 0 kPa) between ramps (Fig. 1c). We avoided high flow regimes (*p*_t_ > 2 kPa) to decrease desiccation and avoid potential damage to the syrinx. When liquid in the bronchi or trachea prevented normal sound production in part of the run, we repeated the entire run. We subjected left and right hemi-syrinx separately in randomized order to this paradigm by allowing air flow only through one side and kept a 1-min rest between runs. The total duration for the experiment was maximally 20 min per individual.

#### Data analysis

Sound was filtered with a 0.1 kHz 3rd order Butterworth high-pass filter, pressure and flow signals were filtered with a 2 kHz 3rd order Butterworth low-pass filter, all with zero-phase shift implementation (filtfilt function, Matlab). Sound, pressure and flow signals were binned in 2 ms duration bins, with a sliding window of 1 ms, and we calculated several parameters per bin. We calculated the root mean square (RMS) values for all signals. For the sound signal, we additionally extracted the fundamental frequency (*f*_o_), source level (SL) and Wiener entropy (WE).

Fundamental frequency was extracted using the yin-algorithm^53^ by manually optimizing minimal aperiodicity (0.05–0.2) and power (0.25–5.0 mPa). Source level at 1 m distance of the emitted sound was defined as:

1 $$\mathrm{SL }= 20\mathrm{log}_{10} \frac{P }{P\mathrm{o}} + 20\;\mathrm{ log}_{{10}}\mathrm{r},$$

where *p* is the RMS of sound pressure (Pa), *p*_o_ is the reference pressure in air of 20 µPa, and r the distance (0.15 m) from the tracheal outlet to the microphone. To calculate WE, we first computed the amplitude spectrum Px of each bin using the periodogram method (Fast Fourier transform (FFT) size = 2048, overlap = 1024 samples). WE was defined as:

2 $$WE={\mathrm{log}}_{10} \left(\frac{geomean(Px)}{mean(Px)}\right).$$

The transmural pressure over the MVM was defined as *p*_t_ = *p*_b_ − *p*_icas_. Phonation threshold pressures (PTP) were identified for *p*_icas_, *p*_b_, and *p*_t_ as the first pressure value for each *p*_b_ ramp where sound power crossed the set threshold. The minimal *f*_o_ was defined as the average *f*_o_ of the first value for each *p*_*b*_ ramp where sound power crossed the set threshold. Minimal SL was defined as the lowest SL value of all *p*_*b*_ ramps per side.

By combining pressure, flow and sound signals we were able to calculate the mechanical efficiency (ME) as the ratio (in dB) of acoustical power (*P*_acoustic_) over aerodynamic power (*P*_aerodynamic_):

3 $$\mathrm{ME }= 10\;\mathrm{ log }_{10} \left( \frac{P_{{\text{acoustic}}}}{P_{{\text{aerodynamic}}}}\right),$$

The acoustic power (W) was assumed to radiate over half a sphere: $P_{{\text{acoustic}}}=\frac{4\mathrm{\pi r}{2}p{2}}{\rho v}$, with air density *ρ* = 1.2 kg m^−3^, speed of sound *v* = 344 m s^−1^, and *p* the RMS sound pressure in Pa. The aerodynamic power *P*_*aerodynamic*_ = *p*_b_*V* where *p*_*b*_ is the bronchial pressure (Pa) and *V* is the flow rate (m^3^ s^−1^). All data analysis was done in Matlab^54^ and R version 3.5.1^55^.

#### MVM dimensions

After the experimental protocol, each syrinx was pinned down in the situ position and kept in 4% (w/v) paraformaldehyde for 24 h at 4 °C. It was then placed in 0.1 M phosphate buffered saline and stored at 4 °C. To ensure that syrinx geometry remained consistent and resembled the position in vivo, we made photos of the geometry in vivo after opening of the ribcage, during in vitro sound production and after pinning and fixation. Additionally, the MVM is structurally supported by the tympanum and cartilages B1-B3 that keep it under base tension in a resting position. This position can be mechanically disturbed, but this would require the bronchi to be pinned in a position or angle clearly outside the in situ range. To quantify the projected size of the MVM of each hemi-syrinx, we carefully cut the syrinx in half (sagittally) and took pictures of MVMs of each hemi-syrinx using a Leica DC425 camera mounted on a stereomicroscope (M165-FC, Leica Microsystems, Switzerland) in Leica Application Suite (LAS Ver. 4.7.0). These photos are made perpendicular to the camera plane because images were sharp in the focal plane. We measured the distances between physiological landmarks in the MVM following^19,27^: (i) the distance between the *medio-ventral cartilage* (MVC) and the *lateral dorsal cartilage* (LDC), and (ii) the distance between the LDC and the *medio-dorsal cartilage* (MDC). Distance measurements between cartilages were taken edge-to-edge in the middle of the cartilage. Additionally, we measured the area of the LDC which is suspended in the MVM. Length and area measurements were taken using ImageJ (Ver. 2.0.0-rc-69/1.52p^56^).

### In vivo sound production

To measure the lowest fundamental frequency produced by an unactuated syrinx in vivo, we performed unilateral tracheosyringeal nerve resections on the right side of the syrinx of juvenile (N = 6) and adult (N = 4) zebra finch males.

#### Tracheosyringeal nerve lesions

Unilateral denervation of the tracheosyringeal nerve was performed as previously described^57^. In brief, birds were anesthetized with isoflurane (induction 3%, maintenance 1.5–2%) and a 5 mm section of the right tracheosyringeal nerve was removed from the trachea through a small incision of the skin. After that the skin was re-sutured and the birds were allowed to recover in a heated cage. Adults were subsequently transferred to holding cages. Juveniles received surgery at 30 DPH and were returned to their parents after surgery. After final song recordings, all animals were sacrificed to confirm the absence of nerve regeneration (adults 20 ± 2 and juveniles 75 ± 3 days post surgery). In all animals we confirmed that the nerve did not grow back.

#### Sound recordings

Song was recorded in custom-built, sound attenuated recording boxes (60 × 95 × 57 cm) and vocalizations were recorded continuously using an omnidirectional microphone (Behringer ECM8000) mounted 12 cm above the cage connected to an amplitude triggered recording software (Sound Analysis Pro^58^). Sound was digitized at a sampling rate of 44.1 kHz with 16-bit resolution (Roland octa capture, amplification 40 dB). Adult birds were recorded several days before and 3 weeks after denervation. Juveniles were recorded at 50 and 100 DPH.

#### Song analysis

Adult birds: For each individual, the most common motif was determined by two experienced observers (IA, CPHE) in recordings acquired before denervation. Subsequently, the same motif was identified in recordings acquired 3 weeks after denervation (days post-surgery: 20 ± 2). A custom written Matlab script was used to extract motifs from sound recordings. 134–200 (average 184 ± 26) motifs per bird were selected for analysis. WAV files were high pass filtered at 0.3 kHz using a 3rd order Butterworth filter.

Syllables or parts thereof were assigned to be produced by the left or right hemi-syrinx based on the following procedure. In zebra finch harmonic stacks, expiratory air flow can be positive on both sides^37,38^, which requires a careful strategy to assign the contribution each side makes during sound production. The two sides can oscillate independently or with some degree of coupling so that they open and close in or out of phase. If these two sides oscillate as an uncoupled system, each side would emit an independent *f*_o_ that would be visible as two simultaneous stacks in the spectrogram. If the *f*_o_s of the oscillators are similar enough, their vibration can become mechanically coupled by energy injections, which would force both sides to oscillate with the same *f*_o_ (i.e., injection locking) or at an integer ratio. For injection locking to occur the *f*_o_s of both oscillators have to be close, which in zebra finches is around 500 Hz. We thus focused on analyzing frequency modulated high *f*_o_ syllables (4–6 kHz) that are spaced far from 500 Hz. If these notes were produced by flow through both sides we would expect to see two independent frequency components after denervating the right side; a frequency modulated high one (left) and an unmodulated low one (right) at the natural resonance of the tissue. If the syllable was produced by the left side only, we would expect to see no change upon unilateral denervation of the right side. If the syllable was produced by the right side only and we abolished tension control of the right side by denervation, we would expect to see a frequency drop to the natural resonance of the tissue of the right oscillator without tension control. Thus, this procedure unambiguously allowed us to assign sides for high notes produced on the right. In line with the literature^41–43^, all high notes in our animals were produced on the right side. For the left side, we analyzed the syllable with the lowest *f*_o_ trajectory that remained unchanged after denervation.

Juvenile birds: Song of juvenile males (N = 6) was analyzed on recordings from 50 DPH (DPH 52.8 ± 1, days post-surgery: 22.3 ± 0.8). At this age, the syllable structure is not fully stereotyped, but motifs usually can be distinguished. We measured the lowest fundamental frequency of 50 motifs per bird irrespective of syllable identity. As the juvenile birds were denervated while singing subsong, we couldn’t distinguish which syllable was produced on which side. Under the assumption that the lowest fundamental is produced by the denervated side, we classified the measured syllables as right produced.

In all birds, frequencies were measured on spectrograms of post denervation recordings (FFT size: 1024, overlap: 75%, Hanning window) in SASlab (Avisoft, Germany). In all juvenile birds the frequency of the lowest harmonic (i.e. *f*_o_) was measured. In all other cases, to increase the *f*_o_ resolution, we measured the 3–5th harmonic and *f*_o_ was calculated as:

4 $${f_{\text{o}}} = {\raise0.7ex\hbox{${{f_n}}$} \!\mathord{\left/ {\vphantom {{{f_n}} n}}\right.\kern-\nulldelimiterspace} \!\lower0.7ex\hbox{$n$}}.$$

where *n* is the nth harmonic.

### Statistics

To assess which pressure (*p*_b_, *p*_icas_, *p*_t_) drives *f*_o_ and SL in adults, we used a delta Bayesian information criterion (ΔBIC) approach. In a first step, we fit a linear model to the *f*_o_-pressure and SL-pressure relationship. We then computed the BIC values for these models and subtracted the BIC of the null model with no pressure variable (*f*_o_ = 1; SL = 1) to calculate the ∆BIC score. The model with the lowest ∆BIC score represents the model with the best fit.

To compare the *f*_o_–*p*_t_ and SL–*p*_b_ relationship over ages and between side and sex, we fit individual linear models to the data and extracted the slopes. For *f*_o_ we split the data in two regions: region S1 (*p*_t_ = 0–0.75 kPa) and region S2 (*p*_t_ = 0.75–2 kPa).

To test whether age, sex or side of the syrinx had a significant effect on our response variables, we fit linear mixed effect models (LMMs) to our data using the lmer function of the lmerTest package^59^ with maximum likelihood optimization. Sex, side and age were fit as fixed effects and animal was included as a random effect to correct for dependence. Model equations can be found in Tables S1, S2 and S3. To determine which effects significantly contribute to the model, we performed model selection using a Likelihood Ratio Test (LRT) with the Chi squared distribution using the lme4 package^60^. The difference between the absolute difference in minimal *f*_o_ between sexes and the difference in minimal *f*_o_ in vivo was assessed with Welch’s t-test. The difference between S1 and S2 slopes for *f*_o_ were assessed using paired t-tests for males and females.

All reported values in the text are mean ± SD. All error bars in figures are SD. The outputs of all LMMs are reported in Supplementary Tables S1, S2, and S3. We chose to present data in figures spilt by sex even when not significant. Statistical significance was accepted at p < 0.05 for all statistical tests.

## Results

### Acoustic output of the adult syrinx

To quantify the functional acoustic output of the adult male and female zebra finch syrinx, we densely sampled the bronchial (*p*_b_) and interclavicular (*p*_icas_) pressure control space in isolated left and right hemi-syrinxes in vitro. For all zebra finch syrinxes tested, sound was produced in a pressure space enclosed by a minimal *p*_b_ and *p*_icas_ and exclusively in the lower half of the *p*_b_, *p*_icas_ space (Fig. 1d). Here a differential or transmural pressure (*p*_t_) exerts force on the MVM that is positive when directed outwards from bronchus to surrounding air sac. We quantified the phonation threshold pressures (PTP) for the three pressures (Fig. 1g). The PTP_b_ was not significantly different for sex (LMM, *p* = 0.418) or side (LMM, *p* = 0.395) and was 1.01 ± 0.28 kPa (range: 0.64–1.68 kPa, N = 11). The PTP_icas_ was also not significantly different for sex (LMM, *p* = 0.638) or side (LMM, *p* = 0.223) and was 0.45 ± 0.27 kPa (range: 0.22–1.07 kPa, N = 11). The PTP_t_ was also not significantly different for sexes (LMM, *p* = 0.057) or side (LMM, *p* = 0.395) and was 0.15 ± 0.19 kPa (range: 4e−5 to 0.75 kPa, N = 11).

Next, we quantified the acoustic output of the adult zebra finch hemi-syrinx within the *p*_b_-*p*_icas_ control space for three parameters: fundamental frequency (*f*_o_), source level (SL) and Wiener entropy (WE). In all animals, fundamental frequency was gradually modulated depending on different combinations of *p*_b_ and *p*_icas_ (Fig. 1d–f). Frequency jumps were not observed. Of all pressures, *p*_t_ described the *f*_o_ data best (∆BIC = − 528, see “Methods”). The continuous, smooth increase of *f*_o_ with *p*_t_ can be clearly seen in Fig. 1e,f. In males, the right typically produced higher frequencies than the left as a function of *p*_t_ (Fig. 1e). However, the minimal *f*_o_ produced was not significantly different and was 511 ± 102 Hz (range: 352–613 Hz, N = 5) and 513 ± 123 Hz (range: 312–625 Hz, N = 5) for left and right hemi-syrinx respectively (Fig. 1h). To describe *f*_o_ as a function of *p*_t_, we split the data in to two regions (Fig. 1e,f); region S1 (*p*_t_ = 0–0.75 kPa) and region S2 (*p*_t_ = 0.75–2 kPa) and fit linear models to the data. The slope of S1 was 189 ± 76 Hz/kPa (range: 67–453 Hz/kPa, N = 5), and was significantly higher than the S2 slope of 55 ± 58 Hz/kPa (range: 25–310 Hz/kPa, N = 5) by 134 ± 18 Hz/kPa (Fig. 1j) (paired t-test, t = 5.3061, df = 9, *p* = 5e−4). In females, the minimal *f*_o_ produced was 529 ± 84 Hz (range: 435–678 Hz, N = 6) for the left and 568 ± 57 Hz (range: 518–650 Hz, N = 6) for the right hemi-syrinx (Fig. 1f). The slope of the S1 region was 181 ± 108 Hz/kPa (range: 67–453 Hz/kPa, N = 6) and was also significantly higher than the S2 slope of 55 ± 58 Hz/Pa (range: − 42 to 167 Hz/kPa, N = 6) by 126 ± 51 Hz/kPa (Fig. 1j) (paired t-test, t = 3.31, df = 10, *p* = 0.0079). Comparing the sexes, the difference in minimal *f*_o_ between left and right seemed smaller in males than in females (Fig. 1i), but this effect was not significant (Welch’s t-test, t = − 1.08, df = 6.42, p = 0.32). The slopes of both S1 and S2 were not significantly different between sex and side and were 185 ± 92 Hz/kPa (range: 25–453 Hz/kPa, N = 11) and 55 ± 58 Hz/kPa (range: − 42 to 167, N = 11), for S1 and S2, respectively (Fig. 1j, Supplementary Table S1).

**Figure 2:**
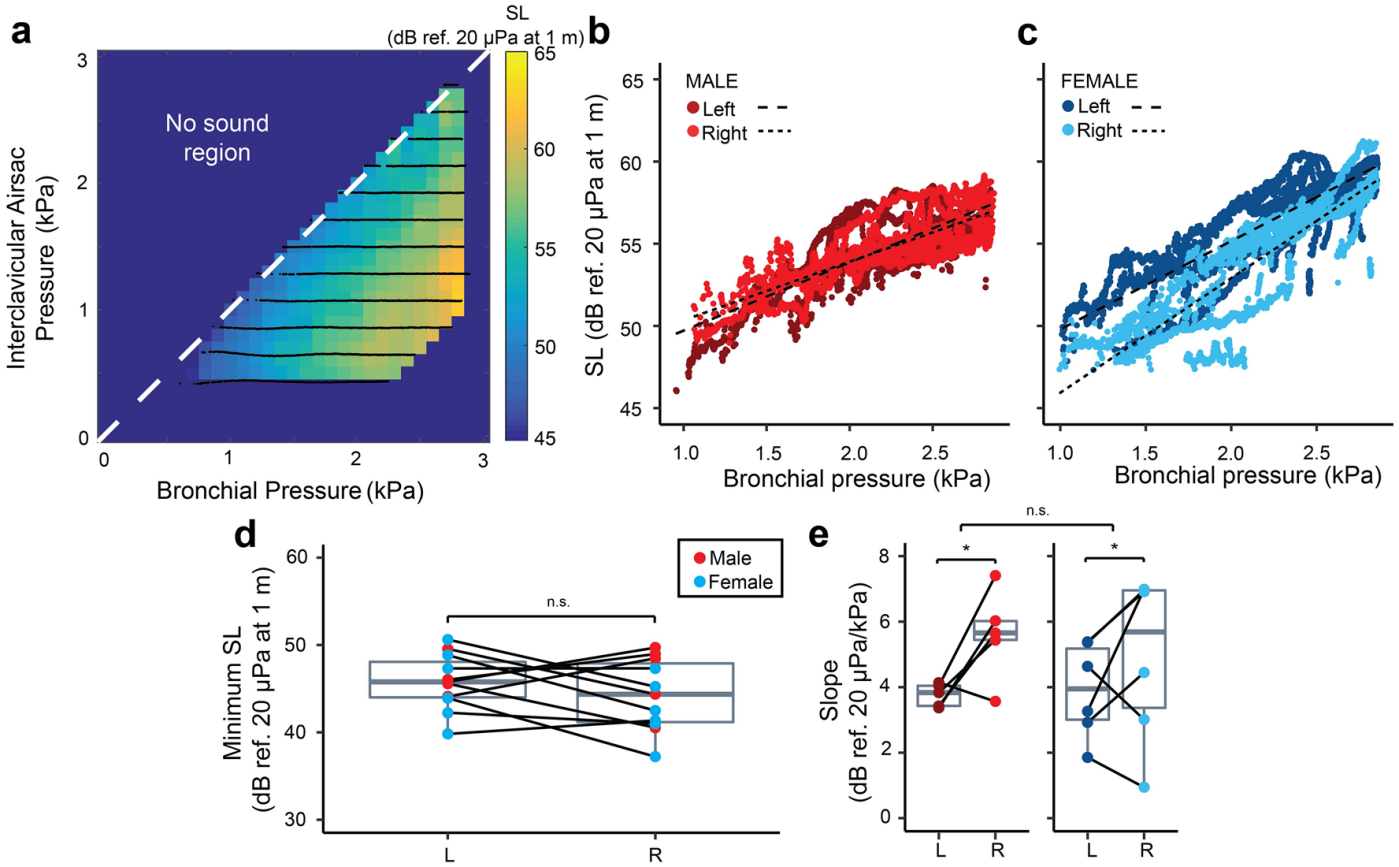
Source level increases with bronchial pressure in the adult zebra finch hemi-syrinx. (**a**) Example of source level (SL) produced in the pressure control space for the left hemi-syrinx of an adult male zebra finch (100 DPH). Source level increases with *p*_*b*_ for both the left (dark red, dark blue) and right hemi-syrinx (light red, light blue) in (**b**) males and (**c**) females, respectively. Black dashed lines indicate linear regressions (Left: long dash; Right: short dash). (**d**) Minimum SL does not differ significantly between the left and right hemi-syrinx or between males and females. (**e**) The SL increase with bronchial pressure was significantly steeper for the right hemi-syrinx than the left hemi-syrinx. For statistics see Supplementary Table S1. *p < 0.05.

Source level at 1 m distance was best described by *p*_b_ (∆BIC = − 78,996) and increased linearly with pressure in both sexes (Fig. 2a–c). The minimum SL (Fig. 2d) was not significantly different for sex (LMM, *p* = 0.149) or side (LMM, *p* = 0.225) and was 45 ± 4 dB re 20 µPa at 1 m (range: 37–51 dB re 20 µPa at 1 m, N = 11). The slope of the SL-*p*_b_ relationship (Fig. 2e) did not differ significantly between males and females (LMM, *p* = 0.704), but was significantly higher on the right side (5 ± 2 dB/kPa, N = 11) than the left side (4 ± 1 dB/kPa, N = 11) (LMM, *p* = 0.016).

**Figure 3:**
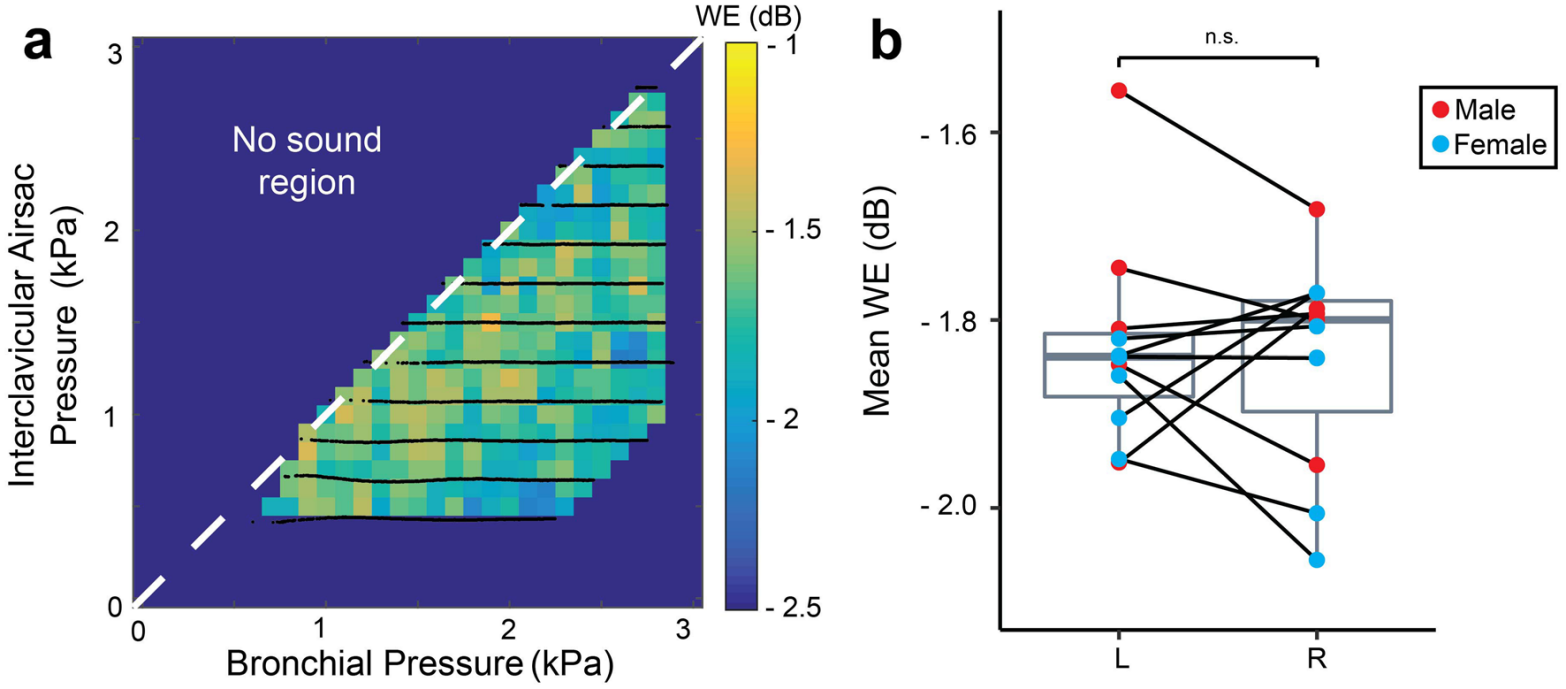
Wiener entropy is not affected by pressure in the adult zebra finch hemi-syrinx. (**a**) Wiener entropy (WE) produced in the pressure control space for the left hemi-syrinx of an adult male zebra finch (100 DPH). (**b**) Mean WE of the left and right hemi-syrinx was not significantly different between male and female zebra finches or for left and right hemi-syrinxes. For statistics see Supplementary Table S1.

We did not show any obvious relation to *p*_b*,*_* p*_icas_, or *p*_t_, and therefore we considered the mean of the entire control space (Fig. 3a). The mean WE did not differ significantly between sexes (LMM, *p* = 0.138) or sides (LMM, *p* = 0.661) and was − 1.8 ± 0.12 dB (range: − 1.6 to − 2.1 dB, N = 11) (Fig. 3b).

**Figure 4:**
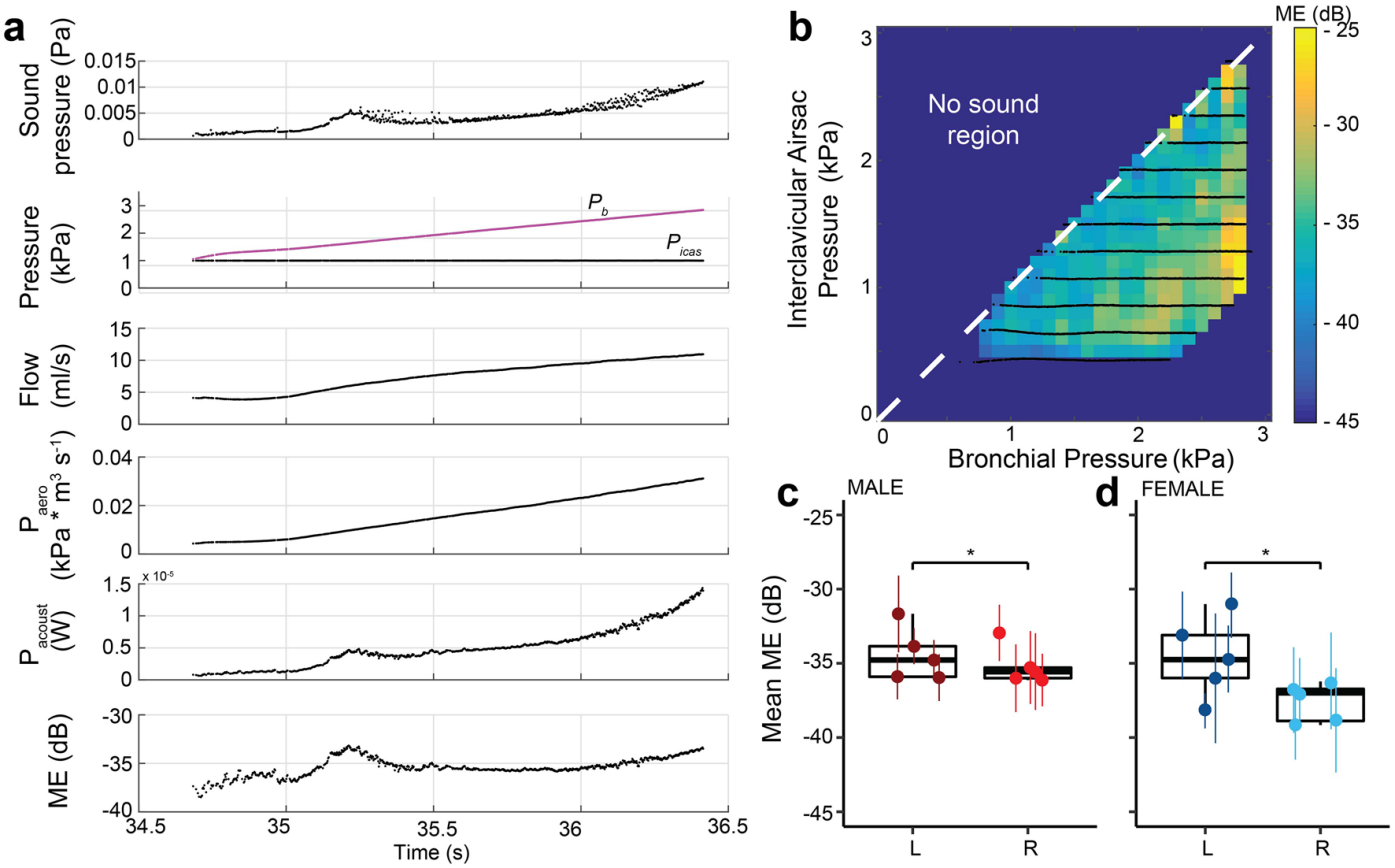
Mechanical efficiency is not affected by respiratory pressure, but is lower on the right side of adult zebra finch. (**a**) Example of one *p*_b_-ramp (from top to bottom) received sound pressure, bronchial and interclavicular air sac pressures, acoustic and aerodynamic power and mechanical efficiency (ME). (**b**) Mechanical efficiency in the pressure control space for the left hemi-syrinx of an adult male zebra finch (100 DPH). (**c**,**d**) Mechanical efficiency was significantly higher for the left hemi-syrinx than the right hemi-syrinx in both sexes. For statistics see Supplementary Table S1. *p < 0.05.

Our setup allowed us to calculate the Mechanical Efficiency (ME) of sound production, which estimates how much of the power in the air flow is transformed into sound (see “Methods”, Fig. 4). We found that ME did not vary systematically with *p*_b*,*_* p*_icas_, or *p*_t_ and therefore considered the mean of the entire control space (Fig. 4b). ME was not significantly different between sexes (LMM, *p* = 0.118), but was significantly lower (LMM, *p* = 0.027) on the right side (− 36 ± 1.8 dB, range: − 39 to − 33, N = 10) compared to the left (− 35 ± 2.2 dB, range; − 38 to − 31 dB, N = 10) (Fig. 4c).

### Acoustic control space does not change over vocal development

**Figure 5:**
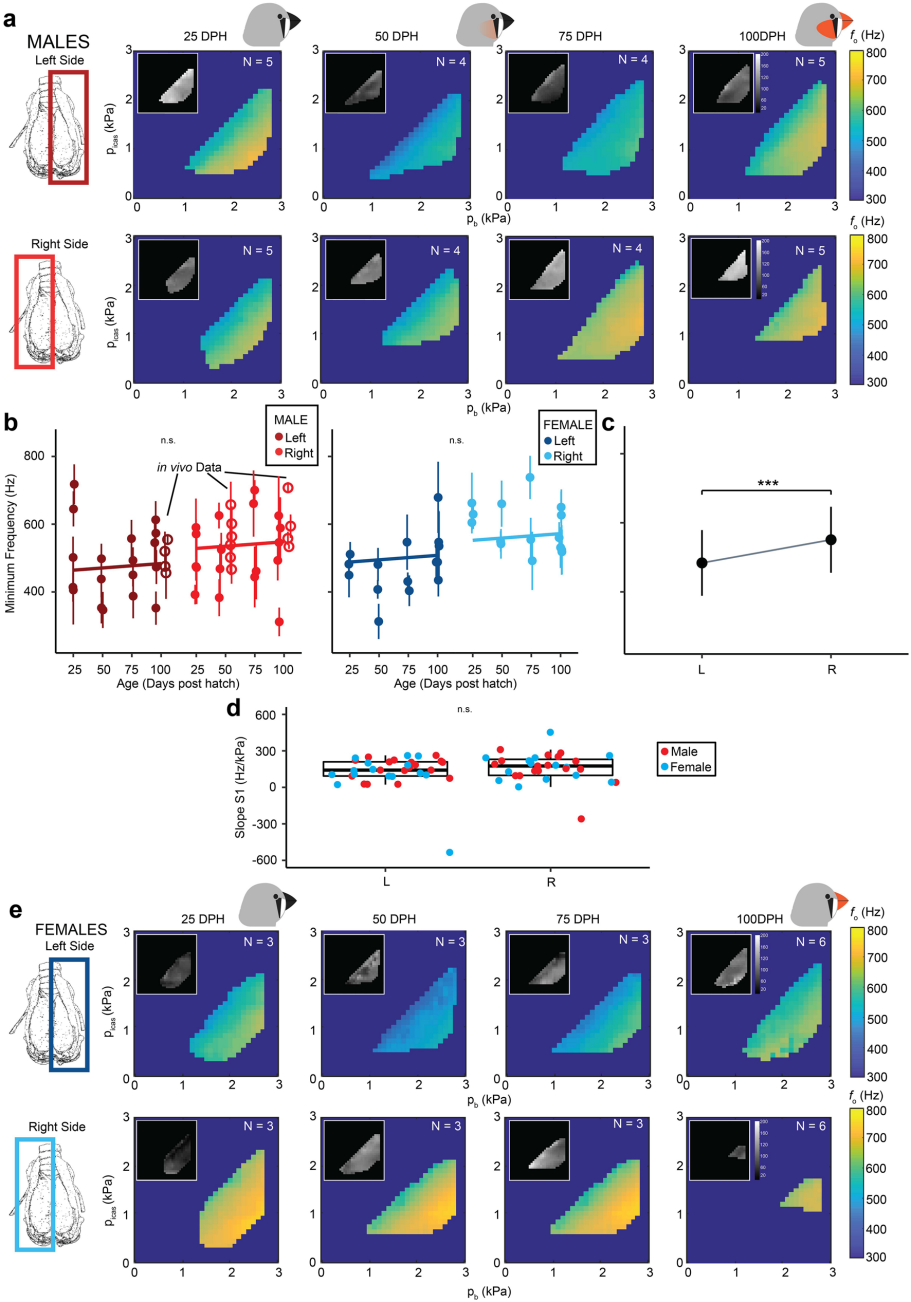
Minimal frequency does not change over zebra finch song development. (**a**) Mean and S.D. (inset) fundamental frequency in the *p*_b_ − *p*_icas_ pressure control space for the left and right hemi-syrinx in males over vocal development. (**b**) Minimum *f*_o_ does not change with age in males and females. (**c**) Minimum frequency was significantly different between the left and right hemi-syrinx, regardless of age or sex. (**d**) Slope S1 for the relationship between *f*_o_ and *p*_t_ was not significantly different for side. (**e**) Fundamental frequency in the *p*_b_ − *p*_icas_ pressure control spaces for the left and right hemi-syrinx in females. For statistics see Supplementary Table S2. ***p < 0.001. Images created with Inkscape 1.0.1 (https://inkscape.org/).

Next, we tested if the output of the isolated zebra finch syrinx changed over vocal development from 25 to 100 DPH (Fig. 5). Like in the adult syrinx, sound was produced exclusively in the lower half of the syringeal *p*_b_, *p*_icas_ space over vocal development. The PTP_b_ was not significantly affected by age (LMM, *p* = 0.257), side (LMM, *p* = 0.268), or sex (LMM, *p* = 0.558) and was 0.9 ± 0.24 kPa (range: 0.53–1.68 kPa, N = 32). PTP_icas_ demonstrated a small but significant increase with age (LMM, *p* = 0.002), but no significant difference between sexes (LMM, *p* = 0.762) or sides (LMM, *p* = 0.274), and went from 0.21 ± 0.8 kPa at 25 DPH (range: 0.006–0.25 kPa, N = 8) to 0.37 ± 0.25 kPa at 100 DPH (range: 0.21–1.1 kPa, N = 11).

Consistently, PTP_t_ also demonstrated a small but significant decrease with age from 0.17 ± 0.08 kPa at 25 DPH (range: 0.06–0.31 kPa, N = 8) to 0.03 ± 0.06 kPa at 100 DPH (range: 4e−5 to 0.16 kPa, N = 11), and marginally, but significantly lower in males (0.0846 ± 0.0981 kPa) compared to females (0.1047 ± 0.0875).

We examined changes in the syringeal *p*_b_-*p*_icas_ control space for three acoustic parameters: fundamental frequency (*f*_o_), source level (SL) and Wiener entropy (WE). Over development, *f*_o_ was also gradually modulated within the *p*_b-_*p*_icas_ space and did not exhibit frequency jumps (Fig. 5). In males and females, the minimal *f*_o_ produced by each hemi-syrinx in vitro did not change significantly from 25 to 100 DPH (Fig. 5b), but the left hemi-syrinx produced a significantly lower minimal *f*_o_ than the right (males: L: 487 ± 106 Hz, N = 18, R: 519 ± 104 Hz, N = 18, females: L: 480 ± 83 Hz, N = 15, R: 590 ± 69 Hz N = 15) (LMM, *p* < 0.001). Interestingly, in 5 out of 18 males the left–right difference was reversed.

The slope of the *f*_o_-*p*_t_ relationship in region S1 did not significantly change with age (LMM, *p* = 0.651), sex (LMM, *p* = 0.630), or side (LMM, *p* = 0.270) and was 91 ± 109 Hz/kPa (range: − 204 to 250 Hz/kPa, N = 32). The slope in region S2 was significantly different between ages (LMM, *p* = 0.0273), but not for sex (LMM, *p* = 0.464), or side (LMM, *p* = 0.474), and was 88 ± 43 Hz/kPa (range: 31–146 Hz/kPa, N = 8), 62 ± 28 Hz/kPa (range: 18–105 Hz/kPa, N = 7), 70 ± 46 Hz/kPa (range: 5–149 Hz/kPa, N = 7), and 65 ± 50 Hz/kPa (range: − 43 to 135, N = 11) for 25, 50, 75, and 100 DPH respectively.

**Figure 6:**
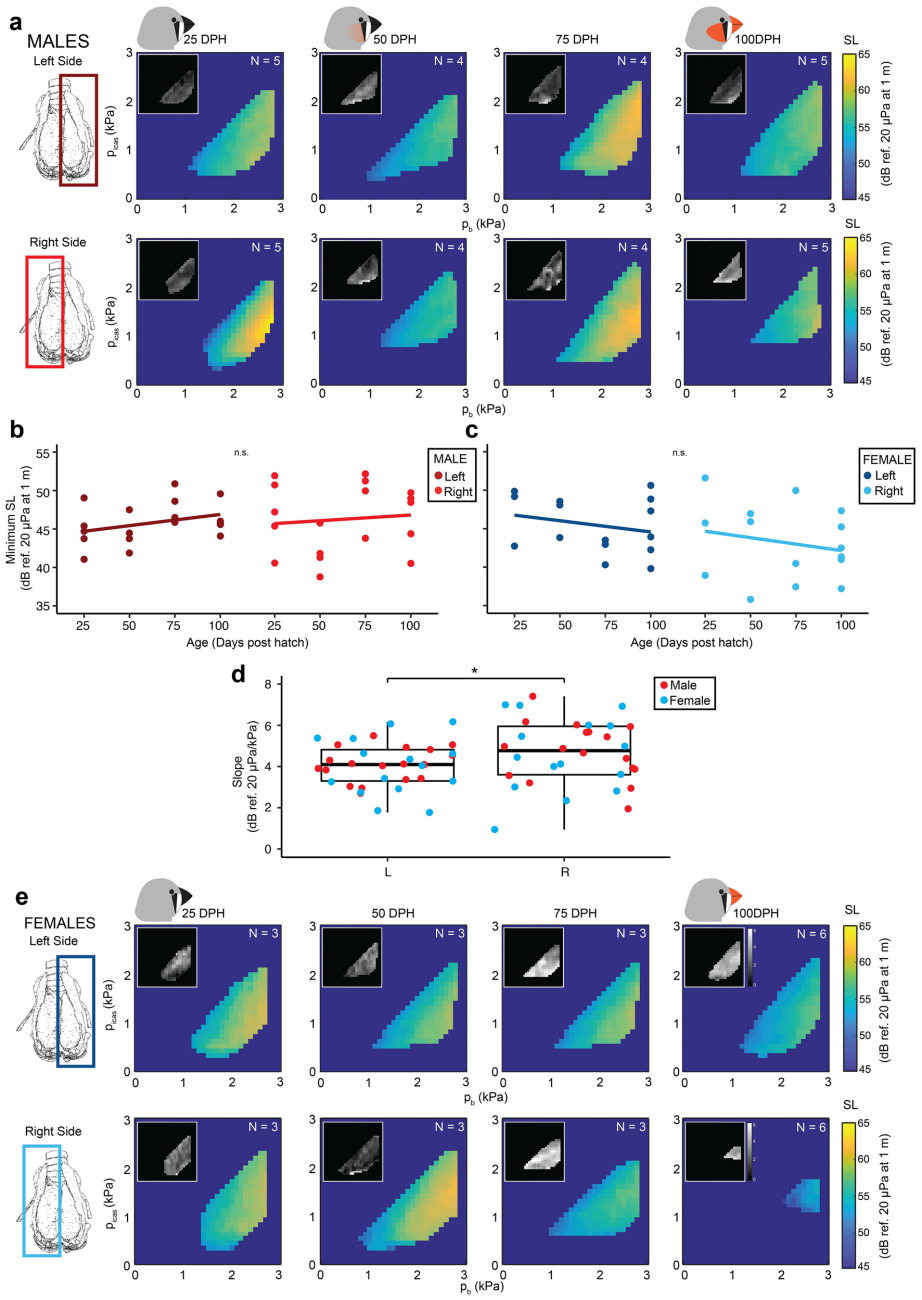
Minimum source level does not change over zebra finch song development. (**a**) Mean SL pressure control spaces in vitro for the left and right hemi-syrinx in males (inset S.D.). Minimum SL produced for (**b**) males and (**c**) females does not change over development, nor was it significantly different between sides. (**d**) Slope for the relationship between SL and *p*_b_ was significantly steeper on the right compared to the left hemi-syrinx. (**e**) Mean SL pressure control spaces in vitro for the left and right hemi-syrinx in females. For statistics see Supplementary Table S2. *p < 0.05. Images created with Inkscape 1.0.1 (https://inkscape.org/).

**Figure 7:**
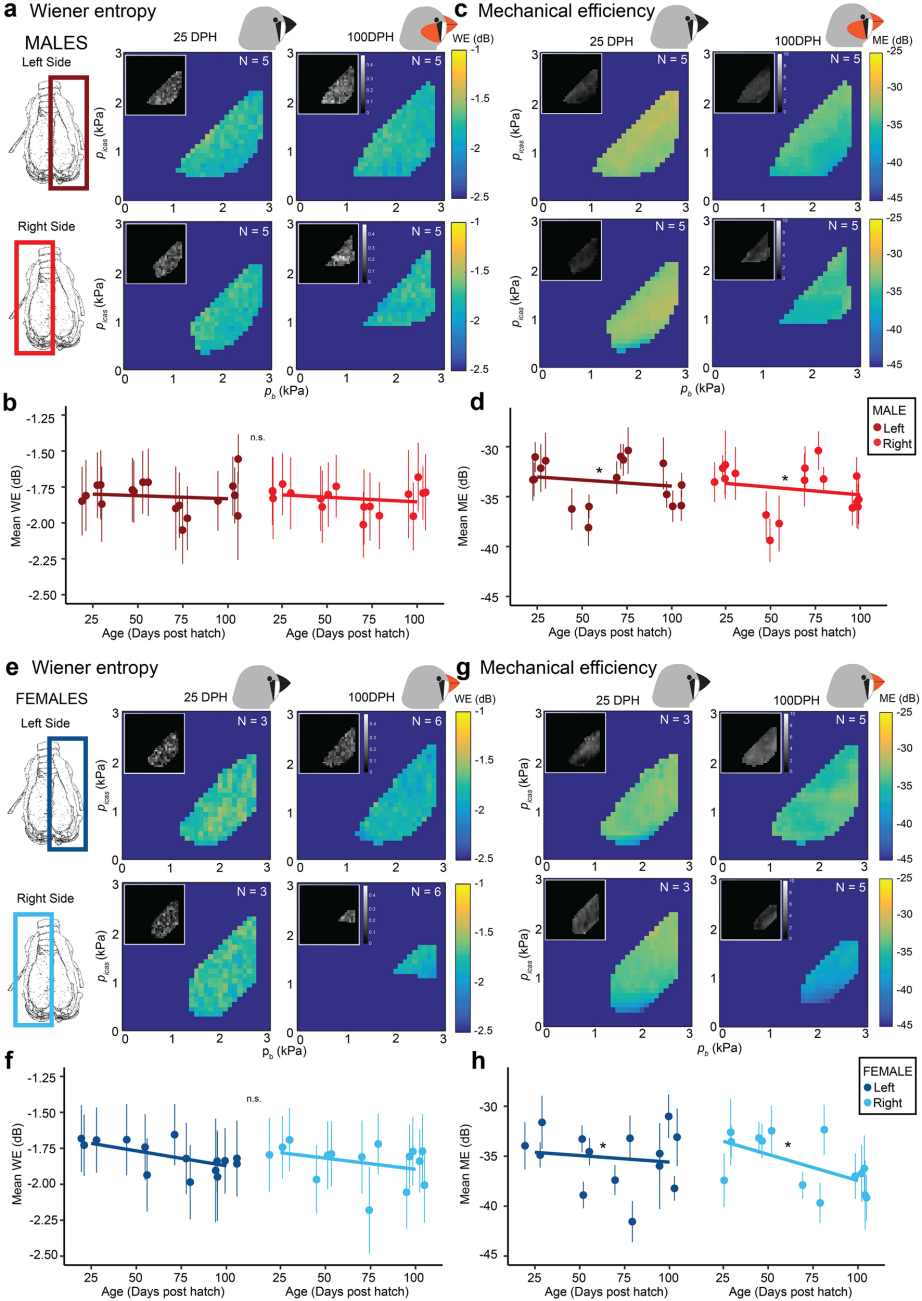
Wiener entropy and mechanical efficiency do not change over zebra finch song development. (**a**) In males, Wiener entropy (WE) for the left and right hemi-syrinx in males age 25 and 100 DPH. (**b**) WE did not change for males over development. (**c**) Mechanical efficiency (ME) for the left and right hemi-syrinx in males age 25 and 100 DPH. (**d**) The mean ME produced for males significantly differed over development. (**e**) In females, WE did not significantly change over development. (**f**) WE did not change for females over development. (**g**) Mechanical efficiency (ME) for the left and right hemi-syrinx in females age 25 and 100 DPH. (**h**) The mean ME of female syrinxes decreased significantly over development. For statistics see Supplementary Table S2. *p < 0.05.

Source level increased with *p*_b_ for all animals (Fig. 6a,e), but the minimal source level did not change significantly with age (LMM, *p* = 0.680), sex (LMM, *p* = 0.374), or side (LMM, *p* = 0.271) and was 44 ± 4 dB with reference sound pressure of 20 µPa at 1 m (range: 36–51 dB, N = 32) (Fig. 6b,c). The SL-*p*_b_ slope also did not change with age (LMM, *p* = 0.83), or sex (LMM, *p* = 0.874), but was significantly less steep (LMM, *p* = 0.037) on the left (4.1 ± 1.0 dB/kPa, range: 1.9–6.2 dB/kPa, N = 32) versus right hemi-syrinx (4.7 ± 1.6 dB/kPa, range: 0.9–7.4 dB/kPa, N = 32) by 0.6 ± 0.3 dB (N = 32) (Fig. 6d). Mean WE did not change significantly for any of the parameters tested (LMM, age: *p* = 0.070, sex: *p* = 0.979, side: *p* = 0.084), and was − 1.8 ± 0.1 dB (range: − 2.1 to − 1.6 dB, N = 32; Fig. 7a–b,e–f).

Next, we calculated the mechanical efficiency (ME) to test if the ability of the syrinx to convert mechanical power to sound changed over song development (Fig. 7c,g). ME did not significantly change with sex (LMM, *p* = 0.091) or side (LMM, *p* = 0.101), but demonstrated a small but significant decrease (LMM, *p* = 0.046) from − 33 ± 1.7 dB (range: − 37 to − 31 dB, N = 8) at 25 DPH to − 35 ± 1.8 dB (range: − 39 to − 33 dB, N = 10) in adults (Fig. 7d,h).

**Figure 8:**
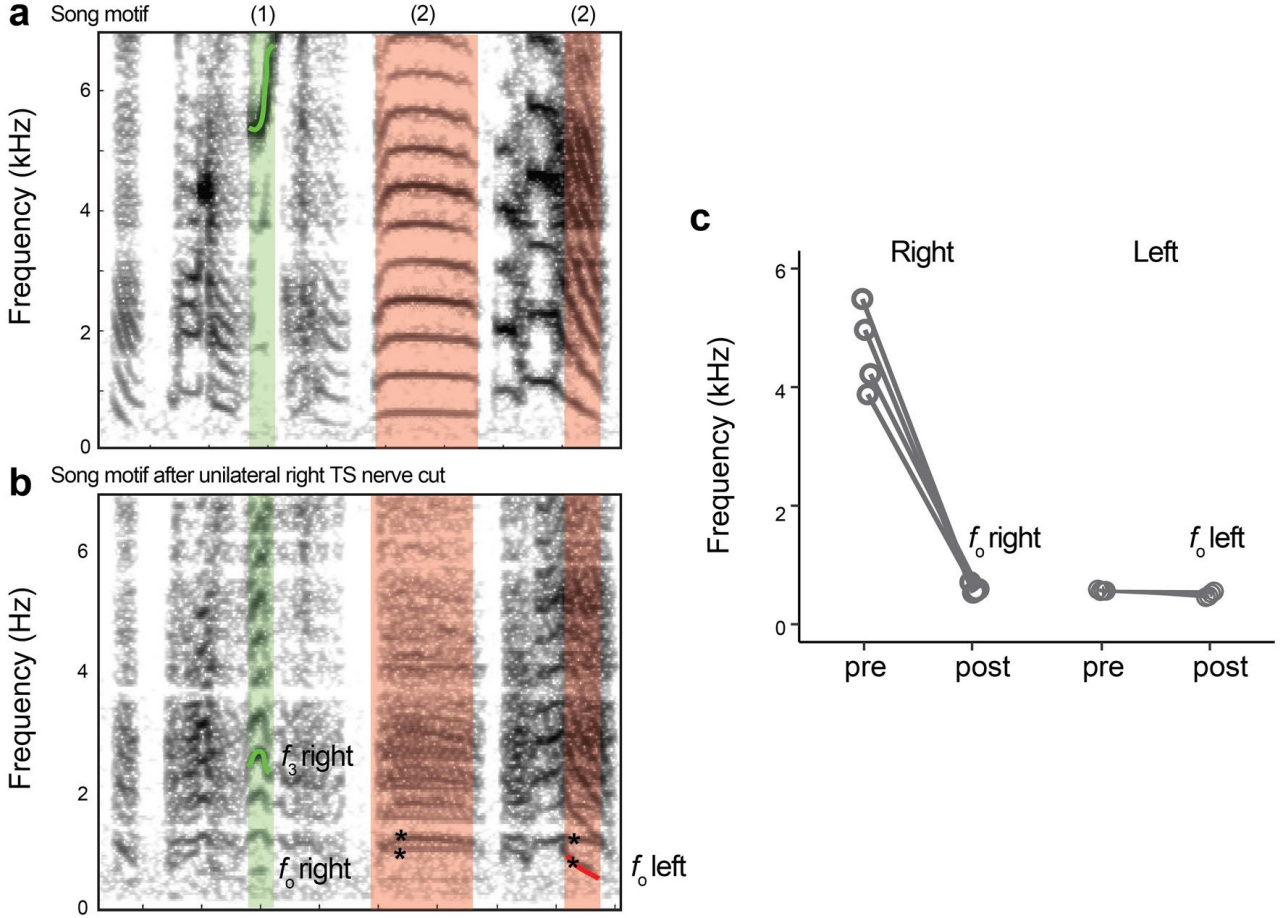
Minimal frequencies produced by the adult left and right hemi-syrinx during song in vivo. Unilateral (right) tracheosyringeal nerve cuts allowed the assignment of left or right produced frequency component in some syllables during song. (**a**) Comparing spectrograms before and (**b**) after nerve cuts on the right side, we observed three types of syllable changes: (1) The *f*_o_ of high frequency (4–6 kHz) syllables decreased (green box). Because we did not observe two simultaneous frequencies in high *f*_o_ notes after nerve cuts, we could reliably assign high notes to be produced by the right hemi-syrinx. (2) In all animals we observed syllables with two simultaneous *f*_o_ trajectories (asterisks) after nerve cuts (orange boxes). This suggested that these syllables were produced by contributions from both sides, and refutes bilateral mechanical injection coupling within those syllables (see “Methods”). Here we could also unambiguously assign the unchanged *f*_o_ time trajectory to the left side. (**c**) Minimal frequencies before and after nerve cut.

Taken together, we observed that the acoustic output of the syrinx control space did not change considerably over development in males and females in vitro. To test whether these in vitro results are representative for the mechanical behavior of the syrinx during in vivo sound production, we measured the acoustic output over development in vivo and used unilateral nerve cuts to exclude the contribution of the syringeal muscles. Because both SL and WE are affected by vocal tract filtering properties, we focused on fundamental frequency. In zebra finches both sides of the syrinx can contribute to syllable production^37,38^. To get around this problem, we developed a procedure to assign the produced syllable or frequency component therein (see “Methods”) to the left or right side. In adults, we observed three types of syllable changes after nerve resection on the right side: (1) The *f*_o_ of high frequency (4–6 kHz) syllables dropped from 4638 ± 725 to 599 ± 77 Hz after denervation on the right side (Fig. 8). We did not observe two simultaneous frequencies in high *f*_o_ notes after nerve cuts. Thus, we could reliably assign high notes to be produced by the right hemi-syrinx only. (2) In all animals we observed syllables with two simultaneous *f*_o_ trajectories after nerve cuts (Fig. 8a). This suggested that these syllables were produced by contributions from both sides, and refuted bilateral mechanical injection locking for those syllables (see “Methods”). In these cases, we could also unambiguously assign side because the unchanged *f*_o_ time trajectory had to be produced by the left side. (3) We also observed syllables whose *f*_o_ trajectories remained unchanged after right nerve cut, which suggested that these syllables were most likely produced on the left. We measured the lowest fundamental frequency produced by males with unilateral syrinx denervation at 50 and 100 DPH (see “Methods”). The minimal *f*_o_ produced by the right hemi-syrinx did not change significantly (Welch’s t-test, t = − 0.91, df = 6.01, p = 0.40) with age and was 555 ± 69 Hz (N = 6) and 599 ± 77 Hz (N = 4) for 50 and 100 DPH, respectively. Furthermore, in adult males the minimal *f*_o_ did not differ between the left (intact) and right (denervated) hemi-syrinx and was 502 ± 44 Hz (N = 4) and 599 ± 77 Hz (N = 4) for left and right, respectively (paired t-test, t = − 2.10, df = 3, p = 0.13). Thus, the in vivo values were consistent with the in vitro data (Fig. 5b), strongly suggesting that the output of the syrinx in vitro is representative of the in vivo situation.

### Morphological changes over development

We lastly investigated if the medial vibratory mass (MVM) dimensions change over development (Figs. S1 and S2). The tension in the MVM can be increased by the shortening of two syringeal muscles that insert on the *medio-ventral cartilage* (MVC) and *medio-dorsal cartilage* (MDC)^20^ (Fig. S1a). A third cartilage, the *lateral dorsal cartilage* (LDC), is embedded within the MVM. A thickening of the tissue between the MVC and LDC is called the medial labium. The two syringeal muscles change the tension of the MVM by changing the distance between (1) MVC and LDC and (2) LDC and MDC (Fig. S1a). The MVC—LDC length did not change with sex (LMM, *p* = 0.72) or age (LMM, *p* = 0.446), but was significantly (LMM, *p* = 0.002) longer on the left (1.13 ± 0.10 mm; range: 965–1297 μm, N = 29) compared to the right (1.05 ± 0.11 mm, range: 798–1363 μm, N = 29) (Fig. S1b). The LDC–MDC length also did not change with sex (LMM, *p* = 0.982) or age (LMM, *p* = 0.191), but it was significantly (LMM, *p* = 0.149) longer on the left (0.71 ± 0.16 mm, range: 387–1107 μm, N = 29) compared to right (0.65 ± 0.13 mm, range: 365–858 μm, N = 29) (Fig. S1c). The area of the LDC was smaller (LMM, *p* < 0.001) on the left (4.1e4 ± 2.2e4 μm^2^, range: 1978–9.9e4 μm^2^, N = 29) compared to the right (6.2e4 ± 3.0e4 μm^2^, range: 11,867–1.1e5 μm^2^, N = 29) hemi-syrinx and increased over development (LMM, *p* = 0.048; Fig. S1d). Lastly, we approximated overall growth of the syrinx as the distance between both ends of the B4 cartilage in the bronchus, and found no significant change for side (LMM, *p* = 0.14), sex (LMM, *p* = 0.17), or age (LMM, *p* = 0.8) and was 1.85 ± 0.23 mm (range: 1280–2196 μm, N = 29; Fig. S1e).

## Discussion

As songbirds learn to sing, both their song system and vocal organ are undergoing postnatal changes that lead to dramatic changes in vocal behavior. Here we show that the acoustic output of the sound generators within the zebra finch syrinx does not change considerably over vocal development. This strongly suggests that the observed acoustic changes during vocal development are caused by changes in the motor control pathway, from song system circuitry to muscle force, and not by changes in the avian analog of the vocal folds. In other words, the properties of the instrument are not changing, but its player is.

Our data present a first detailed quantification of the acoustic output of the isolated zebra finch syrinx when driven by respiratory pressures. We show that both sides of the syrinx, or hemi-syrinxes, produce sound in a well-defined consistent pressure control space. The pressure space is bound by phonation threshold pressures (PTP) for *p*_b_, *p*_icas_ and *p*_t_, which are the same for both sexes and sides. The PTP_b_ values in adults of 1.01 ± 0.28 kPa reported here are consistent with earlier reported PTP_b_ values of 1.2 kPa in situ^61^ and in vivo^48^. The possibility to control the two respiratory pressures (*p*_b_ and *p*_icas_) independently is different from laryngeal systems where no air sacs directly apply force on the vocal folds. To what extent birds can actively and independently control the magnitude of *p*_t_ in vivo during song remains unknown. Certainly, positive pressurization of *p*_icas_ is an essential condition to achieve syringeal vibration in all investigated species in vivo (including zebra finches^45^), and in vitro^14,62,63^, and dynamic *p*_t_ changes up to 1 kPa occur during vocalizations in ringdoves^64–66^. We deliberately covered a larger magnitude pressure range than what the sparse in vivo measurements suggest, ensuring that the in vivo situation is included and forms a subset of the measured parameter ranges presented here.

Our data show that within the investigated control space, source level and fundamental frequency are modulated by respiratory pressures. SL was driven by bronchial pressure, consistent with laryngeal voiced sound production^18^. The SL magnitude of our isolated preparations of 45–60 dB (reference 20 µPa at 1 m) corresponded well to reported SL of zebra finch song in the lab (50–70 dB reference 20 µPa at 1m^67^). Fundamental frequency (*f*_o_) was set predominantly by *p*_t_, consistent with the idea that *p*_t_ acts as a force on the MVM that increases tension^68^, which has also been observed in collapsible tubes^69^. Especially close to the phonation onset, *f*_o_ modulation driven by *p*_t_ is steep (200 Hz/kPa). If a *p*_t_ difference of 1 kPa occurs in zebra finches, this could thus lead to *f*_o_ modulation of 200 Hz. This range is of comparable magnitude to full stimulation of the ventral syringeal muscle that changes *f*_o_ by 200 Hz in zebra finches ex vivo^14^. However, we do not know the magnitude of *p*_t_ during zebra finch song.

In contrast, Wiener Entropy (WE) was not modulated systematically in the pressure space. However, over the course of song learning WE has been shown to change over multiple time scales, from within a day to weeks and months^2,3,70^ and increased entropy variance is linked to better learning success^3,70^. These data suggest that WE is under control of the motor systems of (i) the syrinx, by modulating collision force and thereby spectral slope of the sound source^71^, (ii) the upper vocal tract, by changing the airway resonance properties and thereby frequency content of the radiated sound^72,73^. Additionally, some degree of mechanical coupling between the tissue resonances and vocal tract may occur. In zebra finches the coupling strength will be limited because for most syllables the tissue resonances (500–700 Hz) are far away from vocal tract resonances^74^, and experimental manipulation of tract length does not affect *f*_o_^75^.

Mechanical efficiency may have been an important selecting factor during the evolution of the syrinx as a vocal organ^75^ and our data confirms that the mechanical efficiency of voiced sound production is indeed high in the avian syrinx compared to the mammalian larynx. Our data shows that about 0.05% (− 33 dB) of the flow energy is converted into sound, while in mammals, laryngeal ME is only about 1.10^–3^ to 0.01% (− 50 to − 40 dB) in tigers^76^ and 3.10^–5^ to 1.10^–3^% (− 65 to − 50 dB) in marmosets^6^). ME in the rooster has been reported to be as high as 1.6% in vivo^77^. The ME of the zebra finch in vivo is likely to be higher than we measured in vitro, because our preparation does not include a vocal tract, lacks syringeal motor control, and in vivo both sides can contribute to syllables^37,38^. The reported SL of the zebra finch song in the lab can be up to 70 dB reference 20 µPa at 1 m^67^, which is 10 dB louder than the 60 dB we measured with only respiratory pressure control. A 10 dB SL increase (i.e. root mean square (RMS) is 3 times higher) while maintaining the same aerodynamic power, results in a tenfold increase in radiated acoustic power and a ME of 0.5% (− 23 dB). This is comparable to the very high efficiency reported in roosters^78^. Furthermore, we observed slight differences of ME over development, but these effects were not as pronounced as the changes observed in marmosets, where the adult larynx produces louder and more efficient vocalizations than the infant larynx^6^. Thus, the ME of the zebra finch syrinx is very high already at 25 DPH and remains unaffected by vocal development.

Our data shows that acoustic output of the syrinx is modulated smoothly within the pressure boundaries: both SL and *f*_o_ increased continuously with bronchial and air sac pressures. Thus when driven by physiologically relevant pressures, this nonlinear dynamic system exhibits only one bifurcation from steady (no vibration and no sound) to flow-induced self-sustained vibration, without additional bifurcations, such as period doublings or jumps to deterministic chaos^61,79–82^. Earlier work already showed that in a subset of the pressure control space in situ, additional bifurcations did not occur^61^. Moreover, the supposed bifurcations during song in vivo, e.g. frequency jumps, were shown to not be actual bifurcations^61^, but more likely millisecond scale modulation driven by superfast syringeal muscle^83^. Here we also show that in the independently controlled and densely sampled pressure control space of the zebra finch syrinx in vitro, additional bifurcations do not occur. Thus, driven by physiologically relevant pressures, the acoustic output of the syrinx is continuous, which simplifies sensorimotor control^61^.

Zebra finch song syllables can be produced by MVM oscillations of one hemi-syrinx or by contributions of both sides^37,38^, but we did not study mechanical interaction between the two hemi-syrinxes. However, our data shows that the *f*_o_ magnitude of left and right hemi-syrinx oscillations is about only 50 Hz apart throughout their pressure control space. Because the two sides are also physically close, this raises the possibility that they can become mechanically coupled in vivo by injection locking or pulling. Indeed, abolishing syringeal motor control by bilateral nerve cuts, shows that single *f*_o_ stacks are produced^41–43^, which suggest that the two sides are coupled. Thus, next to controlling acoustic output of each hemi-syrinx, we propose that in songbirds the syringeal muscles may also play an important role in controlling hemi-syrinx coupling strength as a mechanism to control song complexity.

Our data show that the above relations between driving pressures and acoustic output do not change from 25 to 100 DPH over development in zebra finches. The stable acoustic output of the sound generators over development suggests that its mechanical properties are not changing. The *f*_o_ of sound is determined by positioning and tension of and driving pressures on the vocal folds, as well as their resonance properties^19^. Because we show that minimum frequency does not significantly change over development in vitro and in vivo without syringeal control, our data strongly suggest that the effective resonance properties of the syringeal sound generators when driven by physiological realistic pressures do not change over vocal development. Furthermore, the *f*_o_-*p*_t_ relationship (slope S1in Fig. 5d) can be seen as a proxy of MVM stiffness, because *p*_t_ exerts a force on the MVM that causes changes in strain. Since the S1 parameter did not change over development, the change in MVM stiffness as response to strain is also likely changing very little over development. Taken together, the MVM vibration frequencies and response to external force do not change, which strongly suggest that its material properties also do not change over development.

We think it is unlikely this conclusion can be extended to most other songbird species. The adult zebra finch labia are rather homogenous in structure^21^ and we can thus expect a rather isotropic mechanical behavior. However, in the other songbird species investigated, the labia in adults consist of multiple tissue layers^28^ with distinct collagen and elastin fiber orientation and likely anisotropic mechanical tissue properties. In humans, the adult vocal folds also consist of several distinct tissue layers that take over 15 years to developed from a uniform tissue structure at birth to layered after puberty^24–26,84,85^. These layers have different mechanical properties and can contribute differently to mechanical oscillations in humans^85^. Over development even small changes in material properties could drive state changes in the vocalizations of marmosets^6^. Taken together, we think our study emphasizes our general lack of information on tissue maturation over vocal development in songbirds, but even more so, in birds and mammals in general.

We propose that syringeal sound production on the one hand, and its control on the other, are shaped differentially by genetic and environmental factors in zebra finches. First, we propose that the development and maintenance of the sound generators is predominantly under genetic control. Second, we propose that the syringeal muscles and skeletal properties are influenced by use and training, which can ultimately even lead to a sexually dimorphic syrinx.

Our data suggest that MVM mechanical properties are set and maintained mostly by genetic factors and less by usage. First, a salient feature in our dataset is that the adult left hemi-syrinx produces a lower minimal *f*_o_ compared to the right in both sexes, corroborating earlier findings^19^. In most songbirds both hemi-syrinxes contribute to the vocal repertoire^32–37,86,87^ and left–right differences in labial morphology^21,22^ and acoustic output^41,88, 89^ are common in songbirds. In all investigated species to date, except for the Bengalese finch^14,89^ and Australian magpie^90^, the left hemi-syrinx produces lower frequencies. Here we show for the first time that differences between left and right sound generators, size and acoustic output, in zebra finches are already established at 25 DPH. Second, our data suggest that the acoustic output of the MVMs driven by pressure remains stable from 25 DPH. This finding suggests that intrinsic mechanical properties of the bilateral sound generators do not change after 25 DPH, but we would need detailed structural analysis to establish if the MVM tissues indeed have matured. We believe that this latter feature makes the zebra finch a wonderful model system to study vocal development compared to mammalian systems where larynx maturation strongly contributes to vocal output^6,24–26^. Third, the MVM mechanical output seems to remain constant even though they are colliding billions of times over their lifetime and a large variability in use must exist between individuals and sexes. In human vocal folds, strong collisions can lead to several types of lesions that severely affect vocal output^91^. Thus, structural repair of vibratory tissues must lead to maintenance of their properties, but what cellular and molecular mechanisms underlie this maintenance in birds remains unknown. Taken together, these observations support the ideas that the sound generators of the zebra finch syrinx are matured at the onset of vocal learning and that their composition remains stable, which suggests that their composition and dynamical behavior is set predominantly by genetic factors and less by use. The zebra finch syrinx is thus a stable instrument whose postnatal development does not add further complexity to song learning.

In stark contrast, zebra finch syringeal muscles are changing functionally over postnatal development during song learning. Until 25 DPH the syrinx of male and female zebra finches is not clearly distinguishable. However, after 25 DPH, the syringeal muscles start to exhibit sexually dimorphic features, such as increased muscle mass^10^ and speed in males^11^, which leads to a changing transformation from motor neuron spikes to force, often referred to as the neuromuscular transform^13^. Furthermore, muscle force exerted on bones is known to redirect bone deposition and thus can lead to significant bone remodelling^92^. Taken together, we propose the hypothesis that in zebra finches, syringeal muscle activity and resulting forces acting on the syrinx are crucially important drivers to shape the bones and cartilages of the syringeal skeleton. This hypothesis predicts that some anatomical syrinx differences between sexes in zebra finches, where both sexes produce calls but only the males sing, can be caused by muscle use and training driven by the sexually dimorphic brain (i.e., song system). This hypothesis remains to be tested experimentally.

To what extent this idea can be transferred to other (song)bird species also remains to be investigated. In several species sexually dimorphic features of the syrinx have been related to differences in song amount and vocal repertoire^93,94^. For a correlative study, it would be interesting to investigate the syringeal anatomy of songbird species where both sexes produce calls and sing^95^.

## Supplementary Information

Supplementary Information.

## Data Availability

The datasets generated during and/or analyzed during the current study are available from the corresponding author on reasonable request.

## References

[1] Takahashi DY (2015). The developmental dynamics of marmoset monkey vocal production. Science.

[2] Tchernichovski O, Mitra PP, Lints T, Nottebohm F (2001). Dynamics of the vocal imitation process: How a zebra finch learns its song. Science.

[3] Kollmorgen S, Hahnloser RHR, Mante V (2020). Nearest neighbours reveal fast and slow components of motor learning. Nature.

[4] Goldstein MH, King AP, West MJ (2003). Social interaction shapes babbling: Testing parallels between birdsong and speech. Proc. Natl. Acad. Sci. USA..

[5] Oller DK (2013). Functional flexibility of infant vocalization and the emergence of language. Proc. Natl. Acad. Sci. USA..

[6] Zhang YS, Takahashi DY, Liao DA, Ghazanfar AA, Elemans CPH (2019). Vocal state change through laryngeal development. Nat. Commun..

[7] Fee MS, Scharff C (2010). The songbird as a model for the generation and learning of complex sequential behaviors. ILAR J..

[8] Brainard MS, Doupe AJ (2013). Translating birdsong: Songbirds as a model for basic and applied medical research. Annu. Rev. Neurosci..

[9] Darshan R, Wood WE, Peters S, Leblois A, Hansel D (2017). A canonical neural mechanism for behavioral variability. Nat. Commun..

[10] Godsave SF, Lohmann R, Vloet RPM, Gahr M (2002). Androgen receptors in the embryonic zebra finch hindbrain suggest a function for maternal androgens in perihatching survival. J. Comp. Neurol..

[11] Mead AF (2017). Fundamental constraints in synchronous muscle limit superfast motor control in vertebrates. Elife.

[12] Adam, I. & Elemans, C. P. H. Vocal motor performance in Birdsong requires brain-body interaction. *eNeuro* **6**(3) (2019). 10.1523/ENEURO.0053-19.2019 PMC6595438 31182473

[13] Adam I, Elemans CPH (2020). Increasing muscle speed drives changes in the neuromuscular transform of motor commands during postnatal development in songbirds. J. Neurosci..

[14] Elemans CPH (2015). Universal mechanisms of sound production and control in birds and mammals. Nat. Commun..

[15] Jiang W (2020). High-fidelity continuum modeling predicts avian voiced sound production. Proc. Natl. Acad. Sci. USA..

[16] Goller F, Larsen ON (1997). A new mechanism of sound generation in songbirds. Proc. Natl. Acad. Sci..

[17] Mindlin GB, Laje R (2006). The Physics of Birdsong.

[18] Titze IR (2000). Principles of Voice Production.

[19] Fee MS (2002). Measurement of the linear and nonlinear mechanical properties of the oscine syrinx: Implications for function. J. Comp. Physiol. A Neuroethol. Sensory Neural Behav. Physiol..

[20] Düring DN (2013). The songbird syrinx morphome: A three-dimensional, high-resolution, interactive morphological map of the zebra finch vocal organ. BMC Biol..

[21] Riede T, Goller F (2010). Functional morphology of the sound-generating labia in the syrinx of two songbird species. J. Anat..

[22] Riede T, Goller F (2014). Morphological basis for the evolution of acoustic diversity in oscine songbirds. Proc. R. Soc. B Biol. Sci..

[23] Švec JG, Horáček J, Šram F, Veselý J (2000). Resonance properties of the vocal folds: In vivo laryngoscopic investigation of the externally excited laryngeal vibrations. J. Acoust. Soc. Am..

[24] Sato K, Hirano M, Nakashima T (2002). Age-related changes of collagenous fibers in the human vocal fold mucosa. Ann. Otol. Rhinol. Laryngol..

[25] Hammond TH, Gray SD, Butler J, Zhou R, Hammond E (1998). Age- and gender-related elastin distribution changes in human vocal folds. Otolaryngol. Head Neck Surg..

[26] Hammond TH, Gray SD (1997). A study of age- and gender-related elastin distribution changes in human vocal folds. Otolaryngol. Head Neck Surg..

[27] Düring DN, Knörlein BJ, Elemans CPHH (2017). In situ vocal fold properties and pitch prediction by dynamic actuation of the songbird syrinx. Sci. Rep..

[28] Titze I, Riede T, Mau T (2016). Predicting achievable fundamental frequency ranges in vocalization across species. PLoS Comput. Biol..

[29] Mittal R, Erath BD, Plesniak MW (2013). Fluid dynamics of human phonation and speech. Annu. Rev. Fluid Mech..

[30] Andalman AS, Fee MS (2009). A basal ganglia-forebrain circuit in the songbird biases motor output to avoid vocal errors. Proc. Natl. Acad. Sci. USA..

[31] Sober SJ, Brainard MS (2009). Adult birdsong is actively maintained by error correction. Nat. Neurosci..

[32] Suthers RA (1997). Peripheral control and lateralization of birdsong. J. Neurobiol..

[33] Suthers R, Goller F, Pytte C (1999). The neuromuscular control of birdsong. Philos. Trans. R. Soc. B Biol. Sci..

[34] Suthers RA, Zollinger SA (2004). Producing song: The vocal apparatus. Ann. N. Y. Acad. Sci..

[35] Suthers RA (1990). Contributions to birdsong from the left and right sides of the intact syrinx. Nature.

[36] Goller F, Suthers RA (1995). Implications for lateralization of bird song from unilateral gating of bilateral motor patterns. Nature.

[37] Goller F, Cooper BG (2004). Peripheral motor dynamics of song production in the zebra finch. Ann. N. Y. Acad. Sci..

[38] Jensen KK, Cooper BG, Larsen ON, Goller F (2007). Songbirds use pulse tone register in two voices to generate low-frequency sound. Proc. R. Soc. B Biol. Sci..

[39] Yuan, F. Injection-locking techniques for harmonic oscillators. In *Injection-Locking in Mixed-Mode Signal Processing,* 93–133 (Springer, 2020)

[40] Strogatz SH (2018). Nonlinear Dynamics and Chaos: With Applications to Physics, Biology, Chemistry, and Engineering.

[41] Williams H, Crane LA, Hale TK, Esposito MA, Nottebohm F (1992). Right-side dominance for song control in the zebra finch. J. Neurobiol..

[42] Williams H, Cynx J, Nottebohm F (1989). Timbre control in zebra finch (*Taeniopygia guttata*) song syllables. J. Comp. Psychol..

[43] Vallentin D, Long MA (2015). Motor origin of precise synaptic inputs onto forebrain neurons driving a skilled behavior. J. Neurosci..

[44] Gaunt AS, Stein RC, Gaunt SLLL (1973). Pressure and air flow during distress calls of the starling, *Sturnus vulgaris* (Aves;Passeriformes). J. Exp. Zool..

[45] Amador A, Margoliash D (2013). A mechanism for frequency modulation in songbirds shared with humans. J. Neurosci..

[46] Riede T, Fisher JH, Goller F (2010). Sexual dimorphism of the zebra finch syrinx indicates adaptation for high fundamental frequencies in males. PLoS ONE.

[47] Setterwall, C.G. Studier ofver Syrinx hos Polymyoda Passeres. Gleerupska Universitetsbokhandeln, Lund Sweden. pp. 1–128 (1901).

[48] Riede T, Goller F (2010). Peripheral mechanisms for vocal production in birds—Differences and similarities to human speech and singing. Brain Lang..

[49] Goller F, Riede T (2013). Integrative physiology of fundamental frequency control in birds. J. Physiol. Paris.

[50] Srivastava KH, Elemans CPH, Sober SJ (2015). Multifunctional and context-dependent control of vocal acoustics by individual muscles. J. Neurosci..

[51] Elemans CPH (2006). Syringeal muscles fit the trill in ring doves (*Streptopelia risoria* L). J. Exp. Biol..

[52] Daley MA, Goller F (2004). Tracheal length changes during zebra finch song and their possible role in upper vocal tract filtering. J. Neurobiol..

[53] de Cheveigné A, Kawahara H (2002). YIN, a fundamental frequency estimator for speech and music. J. Acoust. Soc. Am..

[54] The Mathworks Inc. MATLAB - MathWorks. http://www.mathworks.com/products/matlab (2016).

[55] R Development Core Team. A Language and Environment for Statistical Computing. (R Found. Stat. Comput., 2018).

[56] Schindelin J (2012). Fiji: An open-source platform for biological-image analysis. Nat. Methods.

[57] Roy A, Mooney R (2007). Auditory plasticity in a basal ganglia-forebrain pathway during decrystallization of adult birdsong. J. Neurosci..

[58] Tchernichovski O, Nottebohm F, Ho CE, Pesaran B, Mitra PP (2000). A procedure for an automated measurement of song similarity. Anim. Behav..

[59] Kuznetsova A, Brockhoff PB, Christensen RHB (2017). lmerTest package: Tests in linear mixed effects models. J. Stat. Softw..

[60] Bates, D., Maechler, M., Bolker, B. & Walker, S. lme4: linear mixed-effects models using S4 classes. R package version 1.1–6. *R* (2014). http://CRAN.R-project.org/package=lme4

[61] Elemans CPH, Laje R, Mindlin GB, Goller F (2010). Smooth operator: Avoidance of subharmonic bifurcations through mechanical mechanisms simplifies song motor control in adult zebra finches. J. Neurosci..

[62] Rüppell W (1933). Physiologie und Akustik der Vogelstimme. J. für Ornithol..

[63] Abs, M. On the Bioacoustics of the Breaking of the Birds Voice. *Zool. Jahrbucher-abteilung fur allg. Zool. Und physiol. Der tiere* **84**, 289–382 (1980).

[64] Gaunt A, Gaunt SL, Casey R (1982). Syringeal mechanics reassessed: Evidence from streptopelia. Auk.

[65] Beckers GJL, Nelson BS, Suthers RA (2004). Vocal-tract filtering by lingual articulation in a parrot. Curr. Biol..

[66] Elemans CPH, Zaccarelli R, Herzel H (2008). Biomechanics and control of vocalization in a non-songbird. J. R. Soc. Interface.

[67] Brumm H, Slater PJB (2006). Animals can vary signal amplitude with receiver distance: Evidence from zebra finch song. Anim. Behav..

[68] Elemans CPHH, Muller M, Larsen ON, Van Leeuwen JL (2009). Amplitude and frequency modulation control of sound production in a mechanical model of the avian syrinx. J. Exp. Biol..

[69] Bertram CD (2004). Flow phenomena in floppy tubes. Contemp. Phys..

[70] Derégnaucourt S, Mitra PP, Fehér O, Pytte C, Tchernichovski O (2005). How sleep affects the developmental learning of bird song. Nature.

[71] Hunter EJ, Titze IR, Alipour F (2004). A three-dimensional model of vocal fold abduction/adduction. J. Acoust. Soc. Am..

[72] Ohms VR, Beckers GJL, ten Cate C, Suthers RA (2012). Vocal tract articulation revisited: The case of the monk parakeet. J. Exp. Biol..

[73] Riede T, Suthers RA, Fletcher NH, Blevins WE (2006). Songbirds tune their vocal tract to the fundamental frequency of their song. Proc. Natl. Acad. Sci..

[74] Ohms, V. R., Ch Snelderwaard, P., ten Cate, C. & L Beckers, G. J. Vocal Tract Articulation in Zebra Finches. 10.1371/journal.pone.0011923 10.1371/journal.pone.0011923 PMC2912855 20689831

[75] Riede T, Thomson SL, Titze IR, Goller F (2019). The evolution of the syrinx: An acoustic theory. PLoS Biol..

[76] Titze IR (2010). Vocal power and pressure-flow relationships in excised tiger larynges. J. Exp. Biol..

[77] Brackenbury JH (1977). Physiological energetics of cock-crow. Nature.

[78] Brackenbury JH (1979). Power capabilities of the avian sound-producing system. J. Exp. Biol.

[79] Wilden I, Tembrock G, Herzel H, Peters G (1998). Subharmonics, biphonation, and deterministic chaos in mammal vocalization. Bioacoustics.

[80] Laje R, Gardner TJ, Mindlin GB (2002). Neuromuscular control of vocalizations in birdsong: A model. Phys. Rev. E Stat. Phys. Plasmas Fluids, Relat. Interdiscip. Top..

[81] Gardner T, Cecchi G, Magnasco M, Laje R, Mindlin GB (2001). Simple motor gestures for birdsongs. Phys. Rev. Lett..

[82] Amador A, Perl YS, Mindlin GB, Margoliash D (2013). Elemental gesture dynamics are encoded by song premotor cortical neurons. Nature.

[83] Elemans CPH, Mead AF, Rome LC, Goller F (2008). Superfast vocal muscles control song production in songbirds. PLoS ONE.

[84] Sato K, Hirano M, Nakashima T (2001). Fine structure of the human newborn and infant vocal fold mucosae. Ann. Otol. Rhinol. Laryngol..

[85] Titze, I. R. *Vocal Fold Physiology: Frontiers in Basic Science* (1993).

[86] Suthers, R. A. How birds sing and why it matters. In *Nature’s Music: The Science of Birdsong* 272–295 (2004) 10.1016/B978-012473070-0/50012-8.

[87] Suthers, R. A., & Zollinger, S. A. From brain to song: the vocal organ and vocal tract. in *Neuroscience of birdsong*, (eds. Zeigler, H.P. & Marler, P.) 78–98 (University Press, Cambridge, 2008).

[88] Nottebohm F, Nottebohm ME (1976). Left hypoglossal dominance in the control of canary and white-crowned sparrow song. J. Comp. Physiol..

[89] Secora KR (2012). Syringeal specialization of frequency control during song production in the bengalese finch (lonchura striata domestica). PLoS ONE.

[90] Suthers RA, Wild JM, Kaplan G (2011). Mechanisms of song production in the Australian magpie. J. Comp. Physiol. A Neuroethol. Sens. Neural Behav. Physiol.

[91] Titze IR, Hunter EJ (2015). Comparison of vocal vibration-dose measures for potential-damage risk criteria. J. Speech Lang. Hear. Res..

[92] Robling AG, Castillo AB, Turner CH (2006). Biomechanical and molecular regulation of bone remodeling. Ann. Rev. Biomed. Eng..

[93] Prince B, Riede T, Goller F (2011). Sexual dimorphism and bilateral asymmetry of syrinx and vocal tract in the European starling (*Sturnus vulgaris*). J. Morphol..

[94] Ballintijn MR, ten Cate C (1997). Sex differences in the vocalizations and syrinx of the collared-dove (*Streptopelia decaocto*). Auk.

[95] Odom KJ, Hall ML, Riebel K, Omland KE, Langmore NE (2014). Female song is widespread and ancestral in songbirds. Nat. Commun..

