# Supplementary material for: Syringeal vocal folds do not have a voice in zebra finch vocal development
Source: Sci Rep. 2021 Mar 19;11:6469. doi: 10.1038/s41598-021-85929-5 (PMC7979720; doi:10.1038/s41598-021-85929-5)
Supplement: Supplementary file 1 — Supplementary Information. [file 41598_2021_85929_MOESM1_ESM.pdf]

Supplementary Information

Syringeal vocal folds do not have a voice in zebra finch vocal development

Alyssa Maxwell, Iris Adam, Pernille S. Larsen, Peter Grove Sørensen, Coen P.H. Elemans

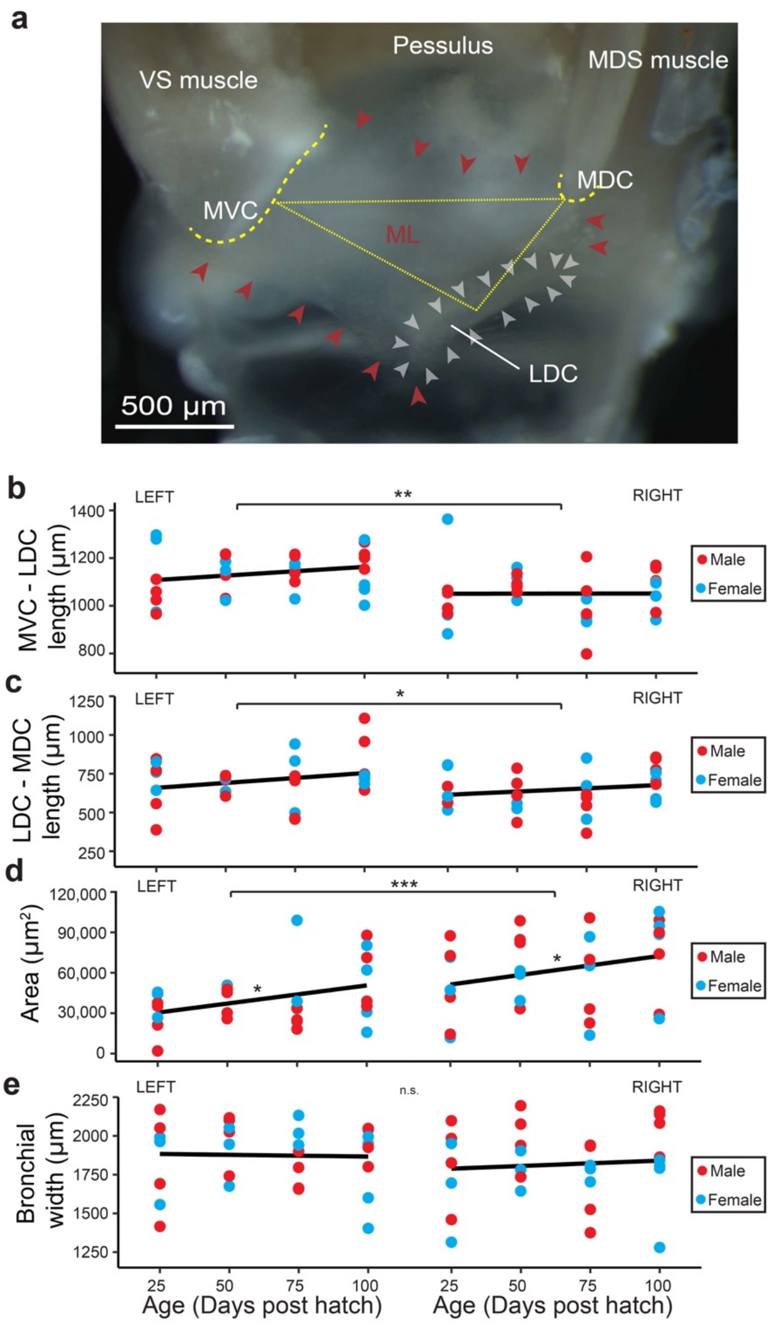

Figure S1. Medial labium dimensions do not change over zebra finch song development in male and female zebra finches. **a)** Anatomical landmarks defining the borders of the Medial Labium (ML, red arrows): the *medio-ventral cartilage* (MVC), the *medio-dorsal cartilage* (MDC) and the *lateral dorsal cartilage* (LDC). The yellow dashed line triangle indicates the position of the measured landmarks. **b)** MVC-LDC length was significantly higher on the left, but did not change over development. **(c)** LDC-MDC length was significantly higher on the left but not over development. **(d)** LDC area for the four investigated age groups was significantly different and differed significantly between the two sides. **(e)** Bronchial width did not significantly differ over development or side. For statistics see Supplementary Table S3. *, p<0.05; **, p<0.01; ***, p<0.001.

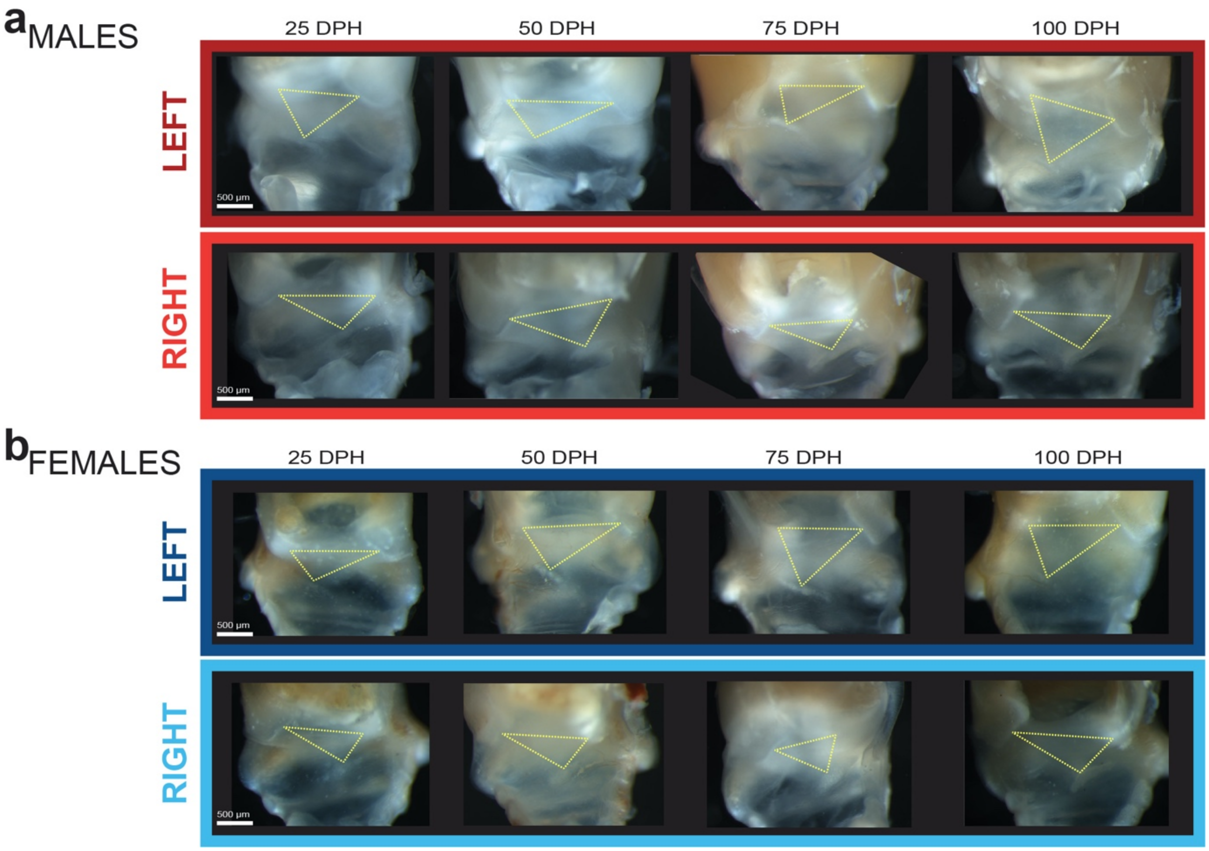

Figure S2. The medial labia over zebra finch song development. Example images of the medial labia of the (a) male and (b) female left and right hemisyrinx over zebra finch song development.

Table S1. Linear mixed effects model outputs for the acoustic results in adult zebra finches.

	Model parameter	Estimate of slope	Std. error	F value	DF	p
	$PTP_b = \beta_0 + \beta_{side} + \beta_{sex} + \epsilon$					
**PTP_b**	Intercept	1.0	0.10			<0.001
	Sex	-0.1	0.12	0.69	1	0.418
	Side	0.1	0.12	0.76	1	0.395
	$PTP_b = \beta_0 + \beta_{side} + \beta_{sex} + \epsilon$					
**PTP_t**	Intercept	0.2	0.06			0.007
	Sex	-0.2	0.07	4.11	1	0.057
	Side	0.1	0.07	0.76	1	0.395
	$PTP_{icas} = \beta_0 + \beta_{side} + \beta_{sex} + \epsilon$					
**PTP_{icas}**	Intercept	0.4	0.10			0.002
	Sex	0.1	0.11	0.23	1	0.638
	Side	0.1	0.11	1.59	1	0.223

	Model parameter	Estimate of slope	Std. error	LL χ^2	DF	p
	$Minf_o = \beta_0 + \beta_{side} + \beta_{sex} + \epsilon$					
**Min f_o**	Intercept	539.6	24.40			<0.001
	Sex	-34.0	35.10	0.90	1	0.34
	Side	21.1	12.40	2.85	1	0.09
	$S1f_o = \beta_0 + \beta_{side} + \beta_{sex} + \epsilon$					
**S1 f_o**	Intercept	172.7	32.62			<0.001
	Sex	17.6	37.59	0.03	1	0.859
	Side	7.2	40.49	0.22	1	0.640
	$S2f_o = \beta_0 + \beta_{side} + \beta_{sex} + \epsilon$					
**S2 f_o**	Intercept	69.0	19.82			0.0021
	Sex	-17.2	23.55	0.27	1	0.606
	Side	-12.3	23.65	0.53	1	0.468

	$MinSL = \beta_0 + \beta_{side} + \beta_{sex} + \epsilon$					
**Min SL**	Intercept	44.7	1.22			<0.001
	Sex	2.4	1.56	2.08	1	0.149
	Side	-1.6	1.24	1.47	1	0.225
	$SlopeSL = \beta_0 + \beta_{side} + \beta_{sex} + \epsilon$					
**Slope SL**	Intercept	3.7	0.58			<0.001
	Sex	0.3	0.78	0.14	1	0.704
	**Side**	**1.4**	**0.50**	**5.79**	**1**	**0.016**

	$MeanWE = \beta_0 + \beta_{side} + \beta_{sex} + \epsilon$					
**Mean WE**	Intercept	-1.9	0.04			<0.001
	Sex	0.1	0.05	2.20	1	0.1383
	Side	0.0	0.03	0.19	1	0.6607

	$MeanME = \beta_0 + \beta_{side} + \beta_{sex} + \epsilon$					
**Mean ME**	Intercept	-35.2	0.69			<0.001
	Sex	1.3	0.79	2.45	1	0.1175
	**Side**	**-1.9**	**0.79**	**4.90**	**1**	**0.0268**

Table S2. Linear mixed effects model outputs for the acoustic results over song development.

	Model parameter	Estimate of slope	Std. error	F value	DF	p
	$PTP_b = \beta_0 + \beta_{age}*age + \beta_{sex} + \beta_{side} + \epsilon$					
**PTP_b**	Intercept	0.812	0.090			<0.001
	Age	0.001	0.001	1.307	1	0.257
	Sex	-0.036	0.061	0.347	1	0.558
	Side	0.067	0.060	1.249	1	0.268
	$PTP_t = \beta_0 + \beta_{age}*age + \beta_{sex} + \beta_{side} + \epsilon$					
**PTP_t**	Intercept	0.368	0.052			<0.001
	**Age**	**-0.002**	**0.001**	**5.287**	**1**	**0.025**
	**Sex**	**-0.095**	**0.035**	**7.212**	**1**	**0.009**
	Side	-0.049	0.035	1.987	1	0.164
	$PTP_{icas} = \beta_0 + \beta_{age}*age + \beta_{sex} + \beta_{side} + \epsilon$					
**PTP_{icas}**	Intercept	0.147	0.066			0.031
	**Age**	**0.003**	**0.001**	**11.083**	**1**	**0.001**
	Sex	0.014	0.045	0.093	1	0.762
	Side	0.049	0.044	1.2E+04	1	0.274

	Model parameter	Estimate of slope	Std. error	LL χ^2	DF	p
	$Minf_o = \beta_0 + \beta_{age}*age + \beta_{sex} + \beta_{side} + \epsilon$					
**Min f_o**	Intercept	482.1	30.3			<0.001
	Age	0.26	0.4	0.4	1	0.510
	Sex	-24	19.1	1.6	1	0.209
	**Side**	**64.14**	**7.0**	**79.0**	**1**	**<0.001**
	$S1f_o = \beta_0 + \beta_{age}*age + \beta_{sex} + \beta_{side} + \epsilon$					
**S1 f_o**	Intercept	102.5	53.8			0.065
	Age	0.3	0.6	0.2	1	0.651
	Sex	18.3	37.9	0.2	1	0.630
	Side	29.6	26.6	1.2	1	0.270
	$S2f_o = \beta_0 + \beta_{age}*age + \beta_{sex} + \beta_{side} + \epsilon$					
**S2 f_o**	Intercept	107.2	17.8			<0.001
	**Age**	**-0.5**	**0.2**	**4.9**	**1**	**0.027**
	Sex	-9.0	12.2	0.5	1	0.463
	Side	-8.0	11.2	0.5	1	0.474
	$MinSL = \beta_0 + \beta_{age}*age + \beta_{sex} + \beta_{side} + \epsilon$					
**Min SL**	Intercept	45.6	1.6			<0.001
	Age	0.0	0.0	0.2	1	0.680
	Sex	1.1	1.1	0.8	1	0.374
	Side	-0.8	0.7	1.2	1	0.271
	$SlopeSL = \beta_0 + \beta_{age}*age + \beta_{sex} + \beta_{side} + \epsilon$					
**Slope SL**	Intercept	4.2	0.6			<0.001
	Age	0.0	0.0	0.0	1	0.830
	Sex	0.1	0.4	0.0	1	0.874
	**Side**	**0.6**	**0.3**	**4.3**	**1**	**0.037**
	$MeanWE = \beta_0 + \beta_{age}*age + \beta_{sex} + \beta_{side} + \epsilon$					
**Mean WE**	Intercept	-1.748	0.045			<0.001
	Age	-0.001	0.001	3.3	1	0.070
	Sex	0.001	0.030	7E-04	1	0.979
	Side	-0.026	0.015	3.0	1	0.084
	$MeanME = \beta_0 + \beta_{age}*age + \beta_{sex} + \beta_{side} + \epsilon$					
**Mean ME**	Intercept	-33.16	1.03			<0.001
	**Age**	**-0.03**	**0.01**	**3.98**	**1**	**0.046**
	Sex	1.24	0.71	2.86	1	0.091
	Side	-0.68	0.41	2.69	1	0.101

Table S3. Linear mixed effects model outputs for MVM morphology.

	Model parameter	Estimate of slope	Std. error	F value	DF	p
	MVC-LDC $= \beta_0 + \beta_{age}{*}age + \beta_{sex} + \beta_{side} + \epsilon$					
**MVC-LDC**	Intercept	1106.5	40.8			<0.001
	Age	0.4	0.5	0.6	1	0.446
	Sex	10.2	28.4	0.1	1	0.720
	**Side**	**-85.7**	**24.9**	**10.0**	**1**	**0.002**
	LDC-MDC $= \beta_0 + \beta_{age}{*}age + \beta_{sex} + \beta_{side} + \epsilon$					
**LDC-MDC**	Intercept	641.8	62.5			<0.001
	Age	1.0	0.8	1.7	1	0.191
	Sex	-1.0	44.9	0.0	1	0.982
	**Side**	**-61.9**	**24.1**	**5.9**	**1**	**0.015**
	LDC Area $= \beta_0 + \beta_{age}{*}age + \beta_{sex} + \beta_{side} + \epsilon$					
**LDC Area**	Intercept	24343.3	10734.9			0.030
	**Age**	**274.2**	**134.1**	**3.9**	**1**	**0.048**
	Sex	-1740.8	7632.1	0.1	1	0.820
	**Side**	**21356.0**	**5022.5**	**14.1**	**1**	**2.0E-04**
	Bronchial Width $= \beta_0 + \beta_{age}{*}age + \beta_{sex} + \beta_{side} + \epsilon$					
**Bronchial Width**	Intercept	1798.5	101.1			<0.001
	Age	0.3	1.3	0.1	1	0.799
	Sex	100.8	72.5	1.9	1	0.172
	Side	-59.4	39.3	2.2	1	0.139
